# Top‐Down Fabricated Wood‐Derived Pressure and Strain Sensors: A Review

**DOI:** 10.1002/advs.202507712

**Published:** 2025-08-07

**Authors:** Yi Ren, Fuyao Liu, Yanan Zhong, Shengbo Ge, Zhipeng Shi, Siling Lin, Hongli Song, Yingying Zhang, Wei Fan

**Affiliations:** ^1^ College of Textile Science and Engineering Key Laboratory of Functional Textile Material and Product of Ministry of Education Xi'an Polytechnic University Xi'an 710048 China; ^2^ Co‐Innovation Center of Efficient Processing and Utilization of Forest Resources College of Materials Science and Engineering Nanjing Forestry University Nanjing 210037 China; ^3^ Department of Chemistry Key Laboratory of Organic Optoelectronics and Molecular Engineering of the Ministry of Education Tsinghua University Beijing 100084 China

**Keywords:** cellulose, pressure sensors, strain sensors, top‐down, wood, wood sponge

## Abstract

As the most abundant and renewable natural resource on earth, wood has been widely used in daily life and industry since ancient times owing to its low cost, facile processability, and environmental sustainability. In recent years, advanced wood‐based materials have rapidly emerged/developed, among which wood‐derived pressure/strain sensors have attracted attention. To date, most reviews on wood‐based sensors have focused on bottom‐up strategies, while relatively few have explored the top‐down approach. However, the top‐down strategies offer notable advantages in terms of process efficiency and reduced energy consumption. This review summarizes recent advances in top‐down fabricated wood‐derived pressure/strain sensors (TWPSS), including raw material selection, the influence of wood composition and microstructure on sensor performance, and top‐down pretreatment methods. Furthermore, the characteristics, sensing mechanisms, and practical applications of TWPSS are analyzed. Finally, current challenges and future research directions are proposed. This review aims to provide insights and serve as a reference for further development of TWPSS.

## Graphical abstract

This review focuses on wood‐derived pressure/strain sensors fabricated via top‐down strategies. It analyzes wood's structural composition, examines processing techniques, discusses sensor types and sensing mechanisms, and reviews existing research. The article concludes with future directions for enhancing performance and scalability.

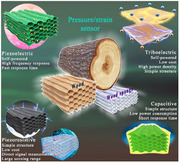

## 1 Introduction

Since the turn of the 21st century, the rapid advancement of Internet of Things (IoT) technology has accelerated the global transition toward digitalization, automation, and intelligent systems.^[^ ^1^ ^]^ Conventional electronic devices, predominantly comprising silicon‐based semiconductors and petroleum‐derived polymers, have played a pivotal role in enabling this transformation. As critical front‐end components for large‐scale data acquisition, pressure and strain sensors are extensively deployed in applications such as smart wearables,^[^ ^2^, ^3^ ^]^ intelligent homes,^[^ ^3^, ^4^ ^]^ transportation systems,^[^ ^5^ ^]^ sports,^[^ ^6^ ^]^ and healthcare,^[^ ^7^ ^]^ significantly enhancing human life. However, the widespread use of these devices has also given rise to pressing environmental concerns.^[^ ^8^, ^9^ ^]^ According to the International Telecommunication Union's (ITU) *Global E‐waste Monitor 2024*, global electronic waste reached an unprecedented 62 million metric tons in 2022, representing an 82% increase since 2010, with no signs of slowing down. Most electronic products are non‐renewable, non‐biodegradable, and contain hazardous components that can infiltrate ecosystems through soil and water, posing long‐term environmental and health threats.^[^ ^8^, ^10^, ^11^, ^12^, ^13^ ^]^ These challenges highlight the urgent need for sustainable material solutions from the source to reduce the environmental footprint of next‐generation electronics.

Wood has emerged as a promising candidate in the ongoing green revolution owing to its inherent renewability, biodegradability, and carbon‐neutral lifecycle—attributes that prevent a net increase in atmospheric CO_2_ emissions.^[^ ^14^, ^15^ ^]^ Crucially, wood possesses a unique, naturally optimized hierarchical structure spanning from the molecular scale (cellulose chains) to the macroscopic scale (cellular porosity, growth rings). Leveraging this inherent architecture for functional devices represents a distinct and innovative approach, often termed the “top‐down” fabrication strategy. This strategy fundamentally differs from conventional processing routes by minimizing structural deconstruction and maximizing the utilization of wood's native framework.

However, conventional approaches predominantly rely on bottom‐up fabrication, where cellulose derivatives—such as cellulose nanocrystals (CNC),^[^ ^16^, ^17^, ^18^ ^]^ cellulose acetate butyrate (CAB),^[^ ^19^ ^]^ cellulose nanofibers (CNF),^[^ ^20^, ^21^ ^]^ or carboxymethyl cellulose (CMC)^[^ ^22^, ^23^, ^24^ ^]^—are extracted from wood through energy‐intensive processes, followed by reassembly into films,^[^ ^25^, ^26^, ^27^ ^]^ hydrogels,^[^ ^28^, ^29^, ^30^ ^]^ or composites.^[^ ^31^, ^32^ ^]^ While enabling precise nanoscale control, these methods inherently dismantle wood's hierarchical structure, incurring high energy costs (e.g., 297.47–5575.9 MJ kg^−1^ for CNC extraction).^[^ ^33^ ^]^

In stark contrast, the top‐down strategy leverages wood's intrinsic multiscale architecture—from molecular cellulose chains to macroscopic porosity—as a naturally optimized template.^[^ ^34^ ^]^ By selectively removing lignin and hemicellulose while preserving the cellulose framework (e.g., via mild delignification), this strategy bypasses energy‐intensive deconstruction and reconstruction steps. Consequently, these top‐down strategies significantly reduce energy consumption (1.83–18.23 MJ kg^−1^).^[^ ^35^ ^]^ This approach offers notable advantages regarding processing simplicity, scalability, and cost‐effectiveness.

Significant progress has been made in recent years in developing wood‐derived pressure and strain sensors using top‐down strategies. To date, most reviews in this field have predominantly focused on bottom‐up strategies for fabricating cellulose‐based sensors.^[^ ^36^, ^37^, ^38^, ^39^, ^40^ ^]^ In this context, this review aims to systematically summarize recent advances in wood‐based pressure and strain sensors developed through top‐down approaches. The influence of wood's intrinsic components and multiscale hierarchical structures on sensing performance is analyzed, while the unique advantages of top‐down strategies in enhancing sensor functionality are highlighted. Innovative applications of these devices in fields such as flexible electronics are also discussed. Furthermore, based on the synthesis of existing research, future research directions are proposed. It is noted that studies involving the direct fabrication of micro‐ and macroscopic morphologies on wood surfaces via processes such as printing or laser sintering to enable sensing functionalities fall outside the scope of this review.

## 2 Wood and Wood Sponge

Lignocellulosic materials have emerged as prominent candidates for advanced sensing applications owing to their renewability, biodegradability, and distinctive structural characteristics. The transformation of natural wood into functional sensing platforms begins with understanding its hierarchical architecture and chemical constituents, as this native structure serves as the fundamental scaffold for subsequent modifications. Balsa wood (*Ochroma pyramidale*) stands out in this context, possessing distinctive properties that render it particularly suitable for sensing applications. The top‐down strategy represents a critical methodology in this transformation process, encompassing the selective removal of specific wood components while preserving the inherent hierarchical organization. Through this meticulous deconstruction process, wood can be converted into a material termed “wood sponge,” characterized by high specific strength, significant anisotropy, and sustainability. These characteristics provide unique advantages for constructing top‐down fabricated wood‐derived pressure/strain sensors leveraging the intrinsic properties of the material.^[^ ^41^ ^]^

### 2.1 Wood Structure and Rationale for Balsa Selection

**Figure 1:**
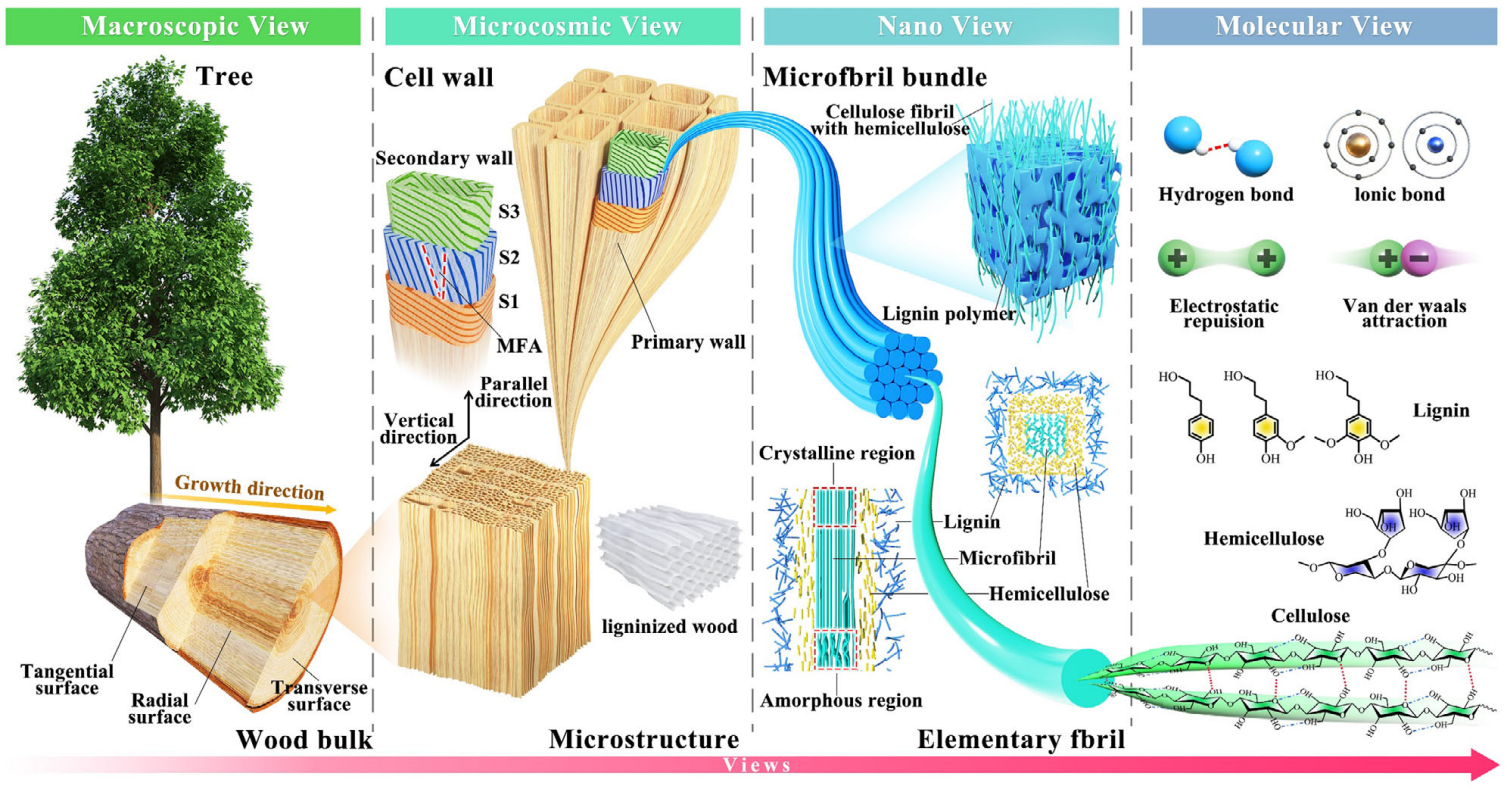
The structure and main components of wood.

The hierarchical structure and chemical composition of wood provide the foundation for sensor design. **Figure** **1** illustrates the multilevel hierarchy and chemical composition of wood. As a natural composite, wood comprises three primary constituents: cellulose (40–50 wt.%), lignin (20–30 wt.%), and hemicellulose (20–30 wt.%).^[^ ^42^ ^]^ Crucially for sensors, cellulose—polymerized from D‐glucose monomers rich in hydroxyl groups via β‐1,4‐glycosidic linkages in linear chains—assembles into elementary fibrils (3–5 nm wide, hundreds of nanometers long) through extensive intermolecular hydrogen bonding and van der Waals interactions. This structure imparts high axial strength.^[^ ^43^, ^44^, ^45^ ^]^ These elementary fibrils aggregate at specific microfibril angles (MFAs) within the secondary cell wall (predominantly the S2 layer,), governing directional mechanics. These aligned microfibrils constitute the primary contributors to the cell wall's mechanical strength.^[^ ^41^, ^46^, ^47^ ^]^ This arrangement results in an anisotropic porous structure featuring axially aligned tracheids (microscale lumens) and nanoscale interfibrillar channels.

Furthermore, the cellulose network enhances material toughness through interfibrillar physical entanglement. Molecular dynamics simulations reveal that dynamic hydrogen bonds—between adjacent cellulose chains, between hemicellulose and cellulose, and between the aromatic rings of lignin and polysaccharides—collectively contribute to wood's stiffness while constraining its deformability.

With the exception of some triboelectric sensors, most TWPSS employ balsa wood as the raw material, owing to several outstanding advantages.

First, its extremely rapid growth rate (reaching 20 meters in height within 5–7 years) results in a loose internal structure (porosity: 74.36–91.54%), thin cell walls (1.52–2.48 µm), and a low density (40–320 kg m^−^^3^). These characteristics facilitate the rapid and uniform penetration of chemical reagents during delignification, enabling efficient and consistent chemical modification throughout the bulk material.^[^ ^48^, ^49^, ^50^ ^]^

**Figure 2:**
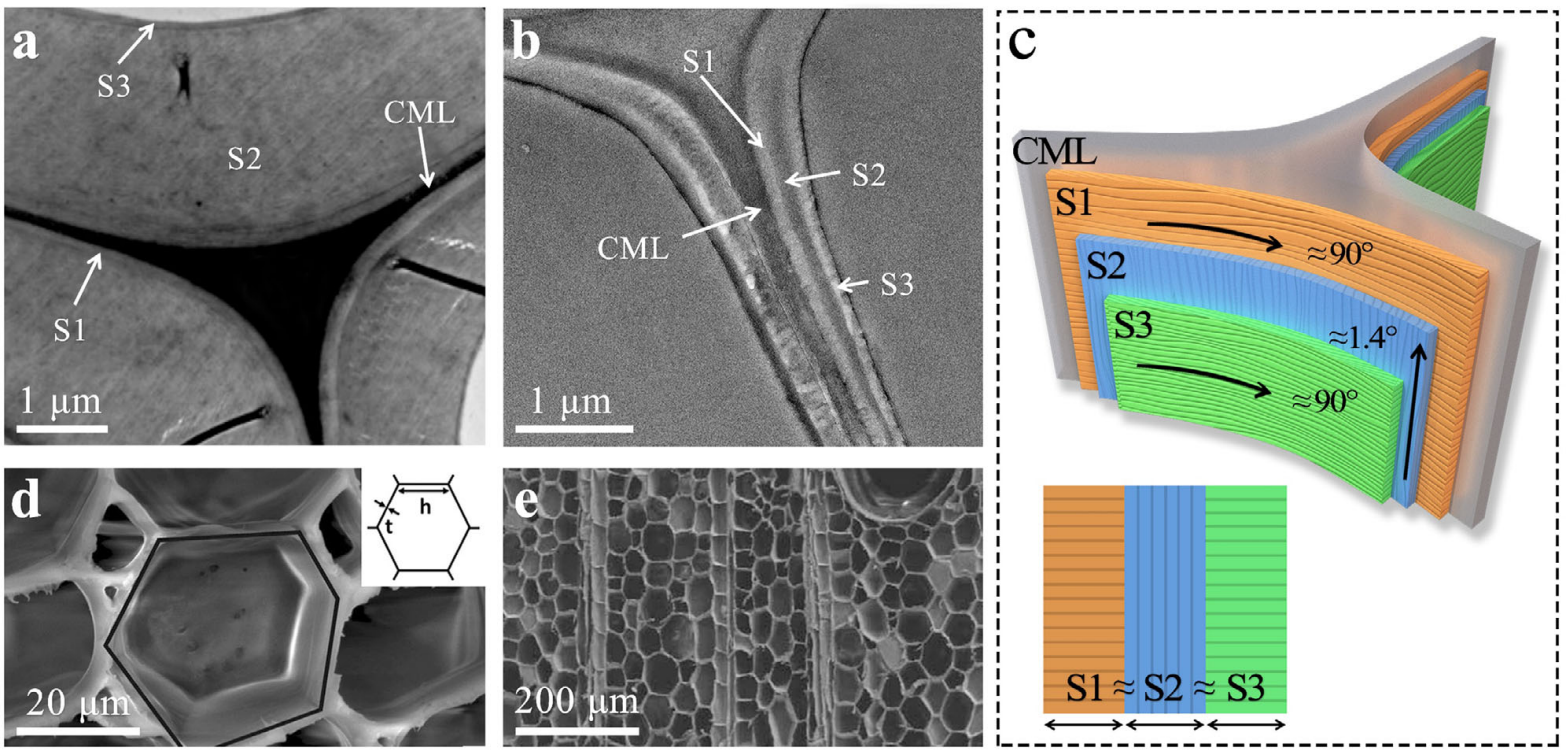
a) TEM image of poplar.^[^ ^56^ ^]^ Copyright 2011, NC State University. b) TEM image of balsa wood.^[^ ^49^ ^]^ Copyright 2015, Springer Nature. c) Balsa wood cell wall simulation model. d,e) SEM images of balsa wood.^[^ ^49^ ^]^ Copyright 2015, Springer Nature.

Second, the S1 and S3 layers are oriented nearly perpendicular to the axial growth direction (MFA ≈ 90°), whereas the S2 layer is highly aligned with the fiber axis (MFA ≈ 1.4°). Moreover, the thicknesses of the S1, S2, and S3 layers are nearly uniform (in contrast to many other woods where the S2 layer dominates, accounting for ≈80–90% of the total thickness). **Figure** **2**a,b show TEM images of poplar and balsa wood cell walls, respectively. Figure 2c provides a schematic of the balsa wood cell wall structure. This orthogonal arrangement and uniform layer thickness substantially enhance the mechanical robustness of the cell wall: the S2 layer primarily provides axial strength, while the S1 and S3 layers contribute transverse reinforcement. Consequently, balsa wood exhibits an extremely high specific strength relative to its low density, with axial compression modulus and strength reaching 6 GPa and 40 MPa, respectively, and flexural modulus and strength reaching 8 GPa and 70 MPa.^[^ ^51^, ^52^, ^53^ ^]^

Furthermore, at the microscopic level, the cellular structure of balsa wood features a highly ordered honeycomb geometry (Figure 2d,e), conferring exceptional stability.

Finally, balsa wood exhibits high cellulose crystallinity (80–90%), which not only reinforces its mechanical integrity but also plays a critical role in its piezoelectric response, directly influencing the performance of piezoelectric TWPSS.^[^ ^54^, ^55^ ^]^

### 2.2 “Top‐Down” Strategies—Delignification

**Figure 3:**
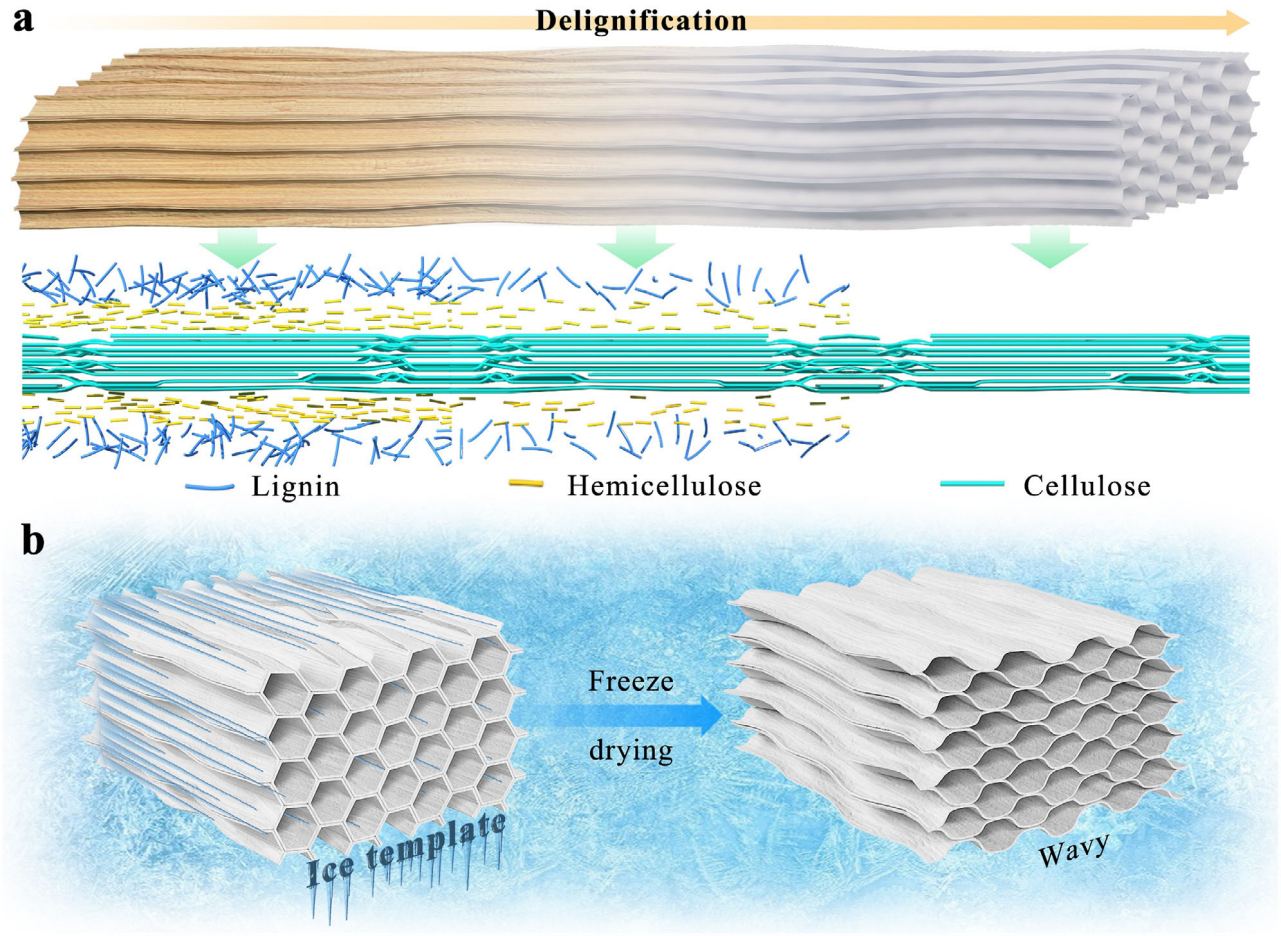
Schematic diagrams of a) delignification, and b) freeze‐drying.

Regardless of the TWPSS type, cellulose invariably plays a pivotal role. The removal of lignin and hemicellulose not only endows wood with excellent compressibility and flexibility (critical for piezoresistive and capacitive sensors) but also exposes the tribo‐positive cellulose (given that lignin and hemicellulose exhibit lower tribo‐positivity), thereby enhancing the material's triboelectric properties. For piezoelectric sensors, delignification increases the overall crystallinity of the cellulose framework, thereby enhancing its output power.^[^ ^57^ ^]^ During this process, lignin and hemicellulose are removed, while the hierarchically anisotropic cellulose framework structure—spanning from the cell wall down to cellulose fibrils and molecular chains—is retained.^[^ ^58^ ^]^ **Figure** **3**a illustrates the stages of delignification.

Various delignification processes have been developed, including enzymatic treatment,^[^ ^59^ ^]^ acid hydrolysis,^[^ ^60^, ^61^ ^]^ alkaline treatment,^[^ ^62^, ^63^, ^64^ ^]^ oxidation treatment,^[^ ^65^, ^66^ ^]^ ionic liquid treatment,^[^ ^67^, ^68^ ^]^ microbial treatment,^[^ ^69^, ^70^ ^]^ deep eutectic solvent (DES) treatment,^[^ ^71^ ^]^ microwave treatment^[^ ^72^ ^]^ and photocatalytic treatment.^[^ ^73^ ^]^ The primary objective of delignification is to disrupt lignin's molecular structure by modifying or cleaving characteristic functional groups (e.g., methoxy and phenolic hydroxyl groups) and key bonds, notably the β‐O‐4 linkages. This decomposes lignin macromolecules into low‐molecular‐weight fragments or enhances their hydrophilicity, thereby facilitating their removal. Concurrently, hemicellulose removal—predominantly achieved through acid hydrolysis, alkaline extraction, or enzymatic action—targets the hydrolysis of glycosidic bonds within polysaccharide chains (e.g., xylan and glucomannan). Ultimately, this process yields a cellulose framework that retains wood's hierarchical structure, termed “wood sponge.”^[^ ^74^, ^75^ ^]^ **Table** **1** summarizes the predominant delignification strategies employed in the fabrication of top‐down wood‐derived sensors. Generally, these strategies can be classified as the NaOH/Na_2_SO_3_ system, the NaClO_2_/CH_3_COOH system, with H_2_O_2_ also frequently used in combination.

**Table 1:** Top‐down pretreatment strategies for TWPSS.

Methods	Delignification solutions	Primary lignin removal mechanism	Primary hemicellulose removal mechanism	Refs.
One‐step	CH_3_COOH/H_2_O_2_ solution	Oxidative cleavage (β‐O‐4 bonds)	Acid hydrolysis	[76]
One‐step	White rot fungus	Enzyme oxidation + Free radical attack	Enzyme hydrolysis	[69]
One‐step	NaOH/Na_2_SO_3_ solution	Nucleophilic sulfonation + alkali hydrolysis fracture (β‐O‐4 bonds)	Alkaline hydrolysis of glycosidic bonds	[77, 78, 79, 80, 81, 82, 83, 84]
One‐step	NaClO_2_ solution (PH is adjusted by CH_3_COOH)	ClO_2_ oxidation + demethoxy ring‐opening	Minor acid hydrolysis	[85, 86, 87, 88, 89, 90, 91, 92, 93, 94]
Two‐step	NaOH solution + NaClO_2_ solution (PH is adjusted by CH_3_COOH)	Alkali swelling + partial β‐O‐4 bonds cleavage	Alkaline hydrolysis of glycosidic bonds (In the first step)	[95, 96]
		Oxidation of ClO_2_ (Ring‐opening remove methoxy group)		
Two‐step	NaClO_2_ solution (PH is adjusted by CH_3_COOH) + NaOH solution	Acid oxidation opens the ring	Alkaline hydrolysis (In the second step)	[97, 98, 99, 100, 101, 102, 103, 104, 105, 106, 107]
		Alkali dissolves oxidation fragments		
Two‐step	NaOH/Na_2_SO_3_ solution + H_2_O_2_ solution	Nucleophilic sulfonation	Alkaline dissolution (In the first step)	[108, 109, 110, 111, 112, 113, 114]
		Oxidation ring‐opening		
Two‐step	KOH_2_ solution + NaClO_2_ solution (PH is adjusted by CH_3_COOH)	Alkali swelling + partial β‐O‐4 bonds cleavage	Alkaline dissolution (In the first step)	[115]
		Oxidation of ClO_2_ (Ring‐opening remove methoxy group)		
Two‐step	NaOH/Na_2_SO_3_ solution + NaClO_2_ solution (PH is adjusted by CH_3_COOH)	Nucleophilic sulfonation + alkali hydrolysis fracture (β‐O‐4 bonds)	Alkaline dissolution (In the first step)	[116, 117, 118]
		Oxidation of ClO_2_ (Ring‐opening remove methoxy group)		
Two‐step	NaClO_2_/CH_3_COONa/CH_3_COOH solution + NaOH solution	ClO_2_ oxidation	Alkaline dissolution (In the second step)	[119]
		Alkali dissolves out fragments		
Two‐step	NaClO_2_ solution (PH is adjusted by CH_3_COOH) + NaOH/Na_2_SO_3_ solution	Acid oxidation opens the ring	Alkaline dissolution (In the second step)	[120]
		Nucleophilic sulfonation + alkali hydrolysis fracture (β‐O‐4 bonds)		
Two‐step	NaClO_2_ solution + NaOH solution	Weak oxidation	Alkaline dissolution (In the second step)	[121]
		partial β‐O‐4 bonds cleavage		

#### 2.2.1 NaOH/Na_2_SO_3_ System

In alkaline conditions, the lignin‐carbohydrate complex (LCC) undergoes cleavage through dual mechanisms. First, OH^−^ cleaves ester linkages between hemicellulose and lignin. Second, SO_3_^2^^−^ undergoes nucleophilic attack at the phenolic hydroxyl groups on lignin's aromatic rings, cleaving methyl‐aryl ether bonds and generating water‐soluble lignosulfonate. Additionally, owing to the strong nucleophilicity of SO_3_^2^^−^, it can also induce cleavage of β‐O‐4 bonds, further facilitating lignin sulfonation. These synergistic effects enhance lignin's solubility and hydrophilicity, enabling its efficient removal.^[^ ^122^, ^123^, ^124^ ^]^

#### 2.2.2 NaClO_2_/CH_3_COOH System

In a buffered environment (pH 4.6) using acetic acid, NaClO_2_ undergoes acid decomposition to produce chlorine dioxide (ClO_2_), a strong oxidant. ClO_2_ preferentially attacks lignin's unsaturated structures, including aromatic rings and olefinic side chains, leading to aromatic ring hydrolysis and side‐chain epoxidation. Ultimately, lignin is degraded into low‐molecular‐weight compounds. In systems containing NaOH, anionic phenols and cleaved β‐ether bonds in lignin are attacked by hypochlorite ions (ClO^−^), forming chlorinated lignin structures. Moreover, the buffered pH 4.6 environment protects cellulose from excessive oxidation.^[^ ^125^, ^126^, ^127^ ^]^ Compared to alkaline treatments, this system minimizes microfibril damage and better preserves the wood's mechanical properties.^[^ ^128^ ^]^ A two‐step combined treatment utilizing NaClO_2_ and Na_2_SO_3_ enables rapid lignin removal. However, this process requires substantial reagent input and generates significant wastewater volumes. Furthermore, the cellulose skeleton can become structurally compromised, leading to significant deterioration of mechanical properties.

#### 2.2.3 H_2_O_2_

As an environmentally friendly chemical, H_2_O_2_ readily generates diverse reactive anions and free radicals. The hydroperoxyl anion (HOO^−^) acts as the primary reactive species, cleaving bonds between aromatic rings and their side chains in lignin. HOO^−^ further degrades unsaturated bonds and aromatic side‐chain groups, such as carbonyl and alkenyl aldehyde structures. These reactions generate cyclic ether intermediates, which subsequently decompose. Concurrently, HOO^−^ attacks aromatic rings, forming epoxide intermediates. These intermediates undergo oxidation and decomposition, yielding low‐molecular‐weight aliphatic compounds, carbonyl derivatives, and carboxylic acids.^[^ ^126^, ^129^ ^]^

#### 2.2.4 White Rot Fungi

Compared to chemical approaches, lignin decomposition mediated by white rot fungi and other microorganisms offers a more environmentally sustainable strategy. This process involves a complex enzymatic system, including: lignin peroxidase (LiP) and manganese peroxidase (MnP), which require H_2_O_2_; laccase (Lac), which uses oxygen (O_2_) as a co‐substrate; and aryl alcohol oxidase (AAO), which generates H_2_O_2_.^[^ ^130^ ^]^ The catalytic activity of these enzymes generates free radicals, primarily aromatic and hydroxyl radicals. These radicals initiate diverse reactions including Cα‐Cβ bond cleavage, β‐O‐4 bond cleavage, aromatic ring cleavage, demethoxylation, and polymerization. However, this biological process requires substantially longer treatment durations compared to chemical methods.^[^ ^131^ ^]^

Based on the treatment process, chemical delignification can be categorized into one‐step and two‐step methods.^[^ ^132^ ^]^ In studies on TWPSS, over 50% of reported cases employ the two‐step delignification method. This approach effectively removes lignin and hemicellulose, enhances the wood sponge's elastic modulus, and significantly improves the sensitivity of piezoresistive and capacitive TWPSS. In contrast, the one‐step method often enables TWPSS to achieve a larger detection range.

### 2.3 Environmental Impact Assessment of Delignification Methods

The environmental sustainability of wood‐derived sensors constitutes a cornerstone of their appeal. Although the top‐down strategy maximizes energy savings by leveraging wood's natural structure, a quantitative assessment of its environmental impact, particularly concerning cellulose extraction, is crucial. **Table** **2** compares the environmental footprints of top‐down and bottom‐up strategies, including energy consumption and global warming potential values.

**Table 2:** Environmental impact of top‐down and bottom‐up strategies.

Method		Electricity (MJ·kg^−1^)	Global warming (kg CO_2_ eq. kg^−1^)	Refs.
Bottom‐up (CNC)	H_2_SO_4_	656.86	36.95	[33]
	DES	636.60	28.33	[33]
	APS	297.47	26.08	[33]
Top‐down (650 kg·m^−3^)	NaClO_2_ solution (PH is adjusted by CH_3_COOH)	18.23	6	[35]
	NaOH/Na_2_SO_3_ solution + H_2_O_2_ solution	7.32	12.86	[35]

Note: “650 kg·m^−3^” indicates the density of the bleached wood templates.

As highlighted in the Introduction, extracting cellulose derivatives such as CNC, CNF, or CMC involves intensive mechanical, chemical, and/or enzymatic treatments to break down the lignocellulosic matrix and isolate nano/micro‐fibrils. In Table 2, the “Electricity” corresponding to “from top to bottom” indicates that the energy consumption of this process is extremely high. This high consumption is primarily attributed to the energy‐intensive processes of high‐pressure homogenization, centrifugation, and drying dilute nanocellulose suspensions. This translates to a significant carbon footprint, primarily associated with grid electricity consumption or process heat generation.

In contrast, top‐down delignification aims to remove lignin and hemicellulose while preserving the macroscopic cellulose skeleton. Methods such as mild alkaline (NaOH/Na_2_SO_3_) or chlorite (NaClO_2_/CH_3_COOH) treatments, followed by washing and freeze‐drying, consume significantly less energy. This order‐of‐magnitude reduction represents the most significant environmental benefit of the top‐down approach, translating to lower greenhouse gas emissions.

Compared to conventional chemicals, deep eutectic solvents (DESs) offer potential advantages including low toxicity, biodegradability, ease of recovery, and reduced water consumption. However, life cycle assessment (LCA) studies on DES applications for top‐down delignification remain limited, warranting further investigation.

Biological methods utilizing white‐rot fungi or specific enzymes (laccases, peroxidases) enable selective lignin removal. Although slower, these processes operate under mild conditions (ambient temperature and pressure, near‐neutral pH), minimizing energy and chemical inputs.

### 2.4 Drying

Commonly used drying methods in the laboratory include atmospheric drying, oven drying, freeze‐drying, critical point drying, and supercritical drying. Among these, freeze‐drying is the predominant choice for TWPSS fabrication (Figure 3b), primarily for two reasons. First, freeze‐dried wood sponges exhibit higher porosity than oven‐dried counterparts prepared from identical wood species. This difference arises because rapid moisture evaporation during oven drying induces shrinkage of the wood sponge's porous network.^[^ ^133^ ^]^ Additionally, rapid drying generates uneven internal moisture gradients, creating localized stress concentrations that can cause warping and cracking.^[^ ^134^ ^]^ Second, freeze‐drying further reduces the Young's modulus of the wood sponge.^[^ ^135^ ^]^ This reduction occurs due to structural modifications within the wood's native pore architecture following delignification. During freeze‐drying, ice formation and expansion within the cell lumen cause physical damage to the cell wall.^[^ ^136^ ^]^ Moreover, these damaged cell walls often adhere to adjacent rays under the ice template, forming a layered structure with multiple stacks and connections. This architecture enhances the radial compressibility of the wood sponge.^[^ ^108^ ^]^

### 2.5 Wood Sponge Structure‐Function Relationships

Wood sponge is a delignified cellulose framework derived from wood through chemical or biological treatment. This top‐down subtractive modification strategy circumvents the complexity of fiber reassembly, preserving natural wood's intrinsic, hierarchical 3D fiber architecture.^[^ ^58^, ^137^ ^]^ By extensively removing the matrix components—primarily lignin and hemicellulose—this process increases the internal pore volume and surface area, significantly enhancing the accessibility of cellulose microfibrils for functional additives and chemical modifications.^[^ ^125^ ^]^ Mechanically, the disruption of dynamic hydrogen bonding within and between cellulose and hemicellulose chains, coupled with the transformation from a native stress‐concentrated honeycomb microstructure to a lamellar architecture exhibiting more uniform stress distribution, imparts superior elastic deformation capacity to the wood sponge. This architecture prevents localized collapse during compression and facilitates rapid shape recovery upon load removal. Consequently, the material exhibits excellent fatigue resistance under high‐cycle compression (>10 000 cycles). The mechanical performance is also strongly influenced by the delignification strategy, which governs the extent of lignin and hemicellulose removal. The radial tensile strength correlates with the remaining lignin and hemicellulose content within individual fibers. As lignin is removed, the proximity and aggregation of cellulose fibrils increase, tightening interfibrillar connections and enhancing tensile strength. In contrast, hemicellulose removal results in a looser fibril arrangement, compromising mechanical integrity.^[^ ^138^ ^]^

Wood sponge also retains the pronounced anisotropy of its natural wood, manifested through its mechanical behavior, ion transport efficiency, and thermal conductivity. This anisotropy stems from the highly ordered alignment of cellulose microfibrils, with an orientation index reaching up to 0.84, far surpassing that of cellulose nanofiber or nanocrystal aerogels fabricated via freeze‐casting.^[^ ^58^ ^]^ In terms of ion transport, delignification introduces numerous nanoscale channels between separated cellulose fibrils, facilitating efficient multiphase transport of ions, molecules, gases, liquids, and particles.^[^ ^139^, ^140^, ^141^ ^]^ The continuous axial alignment of the cellulose network further promotes rapid transport, a critical feature for hydrogel TWPSS.^[^ ^142^ ^]^ Although there are abundant tiny pores between the cell walls in the radial direction, the cell walls separate the channels of the wood, and the lack of ion transport pathways hinders the ion transport to a certain extent.^[^ ^57^ ^]^

## 3 Top‐Down Fabricated Wood‐Derived Pressure and Strain Sensor

Pressure/strain sensors detect applied forces or deformations, which cause changes in electrical properties (e.g., resistance, capacitance, voltage).^[^ ^143^ ^]^ Depending on the different mechanisms underlying electrical signal changes, TWPSS can be classified into four types: piezoresistive, capacitive, triboelectric, and piezoelectric. Their typical structure consists of a top substrate layer, a bottom substrate layer, at least one active sensing layer, and an electrode layer. In TWPSS, wood often simultaneously functions as the substrate and the active layer. This section integrates wood's compositional and structural features to elucidate the working principles of these four sensor types and analyze the unique advantages of wood‐derived materials in fabricating pressure/strain sensors.

Meanwhile, this section reviews the existing research on top‐down fabricated wood‐derived pressure/strain sensors, focusing on the preparation strategies, structural designs, and key performance indicators, including sensitivity, operating range, and linearity.

### 3.1 Piezoresistive Sensor

**Figure 4:**
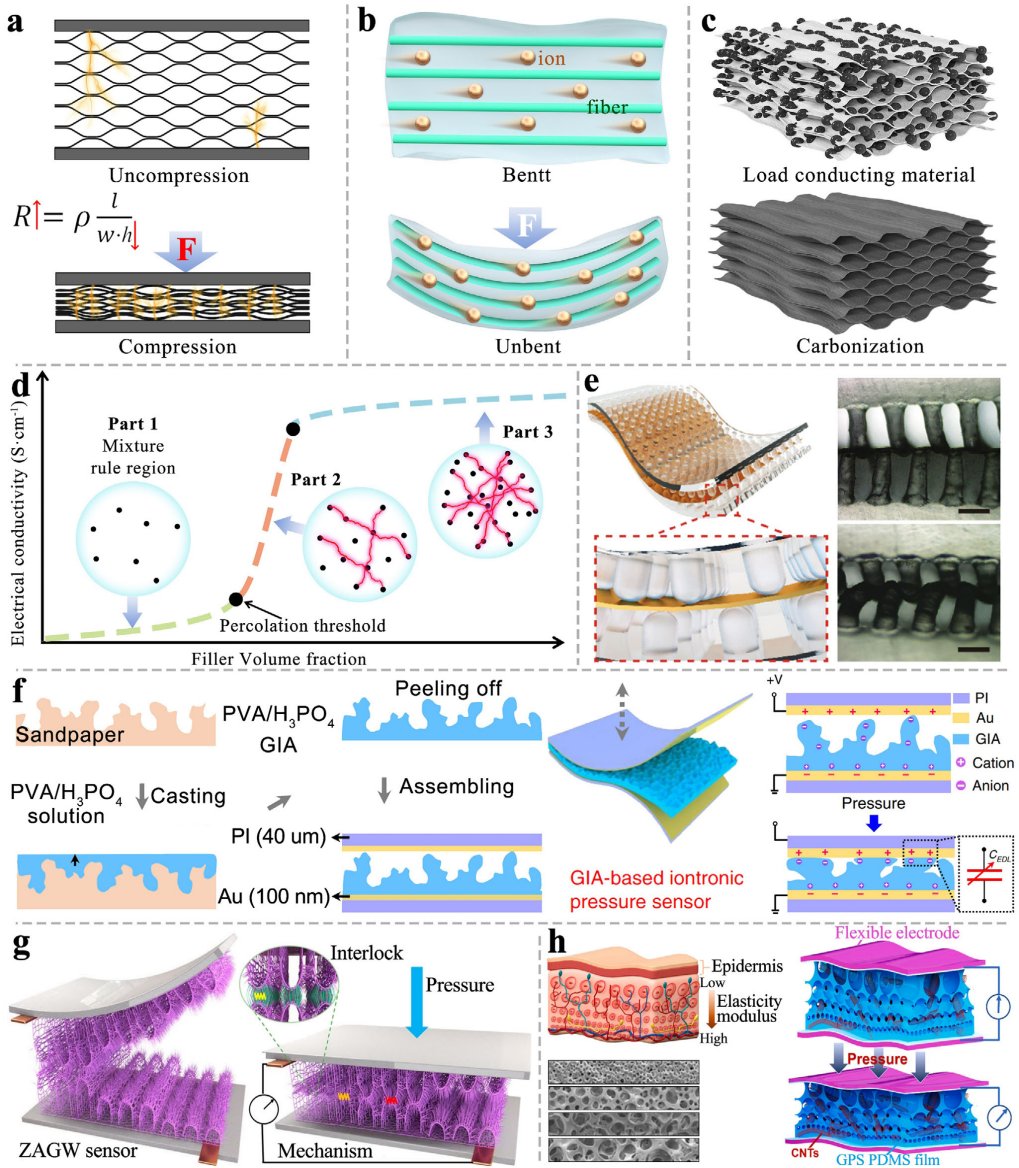
Sensing principles of piezoresistive TWPSS. a) Sensing mechanism of piezoresistive aerogel. b) Sensing mechanism of piezoresistive hydrogel. c) Wood sponge loaded with conductive particles and carbonized. d) Relationship between the volume content of conductive filler and the electrical conductivity of the gel. e) The cylindrical microstructures enhance the sensitivity of piezoresistive sensors. Reproduced with permission.^[^ ^146^ ^]^ Copyright 2024, Wiley‐VCH. f) A pressure sensor with a rough surface structure was fabricated using sandpaper as a template to improve its sensing performance. Reproduced with permission.^[^ ^148^ ^]^ Copyright 2020, Wiley‐VCH. g) Gradient wrinkling of electrospun film is used to achieve large sensitivities and broad sensing ranges. Reproduced with permission.^[^ ^152^ ^]^ Copyright 2024, Wiley‐VCH. h) Inspired by the structure and functions of the human fingertip, fingerprint‐like patterns and interlocked microstructures are used for piezoresistive sensing. Reproduced with permission.^[^ ^149^ ^]^ Copyright 2022, Elsevier Ltd.

Piezoresistive sensors are widely used in thin TWPSS due to their simple structure and easy fabrication. Their operating principle relies on the material's resistance response to external mechanical stimuli, which involves two primary mechanisms: geometric and physical piezoresistive effects. Based on these mechanisms, piezoresistive TWPSS are generally divided into two types: aerogel‐based (**Figure** **4**a) and hydrogel‐based (Figure 4b), each with distinct sensing characteristics and underlying mechanisms.

#### 3.1.1 Principle of Piezoresistive Sensor (Aerogel)

The geometric piezoresistive effect exhibited by aerogels is usually controlled by two mechanisms. The first involves a change in the contact area between the active sensing layer and the electrode under applied pressure. The second relates to variations in the internal conductive network within the sensing material. The former mechanism is highly dependent on the microstructural features of both the sensing layer and the electrode surface. These microstructures are commonly fabricated by casting polymer substrates onto pre‐patterned templates, followed by curing. The templates can be laser‐engraved patterns (Figure 4f),^[^ ^144^ ^]^ natural leaves/petals,^[^ ^145^ ^]^ sandpaper (Figure 4d),^[^ ^146^ ^]^ porous polystyrene,^[^ ^147^ ^]^ 3D‐printed molds,^[^ ^148^ ^]^ etc. The materials of the active layer are mostly high‐molecular polymers, and common structures include pyramid‐shaped, cone‐shaped, dome‐shaped, column‐shaped, etc.^[^ ^149^ ^]^ Ma et al. proposed a pressure sensor based on a gradient wrinkled electrospun polyurethane membrane with MXene‐embedded ZnO nanowire arrays (Figure 4e).^[^ ^150^ ^]^ However, wood—as a natural anisotropic material—can also be modified to exhibit microstructured surfaces via mechanical sawing, though achieving precise and reproducible patterns remains challenging.^[^ ^151^ ^]^

The second mechanism, variations in the internal conductive pathways, is primarily dictated by the elastic deformation of the active sensing layer. Upon external force application, the material compresses, increasing the number of conductive pathways between electrodes (Figure 4b) and thereby reducing the overall resistance of the system.^[^ ^152^, ^153^ ^]^ This mechanism entails two key criteria: the active layer must exhibit sufficient elastic deformability and possess electrical conductivity. Delignified wood sponge, with its high compressibility, meets the requirement for mechanical flexibility. Electrical conductivity can be introduced either through carbonization or the incorporation of conductive fillers (e.g., MXene, CNTs) (Figure 4c). However, carbonization may compromise the material's long‐term mechanical resilience and fatigue resistance due to irreversible microstructural damage and diminished elastic recovery occur under cyclic loading. This sensing behavior is particularly relevant to aerogel TWPSS, where sensing relies predominantly on macroscopic geometric deformation of the active layer in response to external pressure, specifically, changes in the radial thickness of the wood matrix—classified as the geometric piezoresistive effect. The fundamental relationship governing resistance in such pressure sensors is expressed as Equation (1):

(1) $$R=\rho \frac{l}{w\cdot h}$$

where *R* is the resistance of the material, ρ is the resistivity, and *l*, *w*, and *h* are the length, width, and thickness. When the sensor is under pressure, it mainly corresponds to the change of the thickness *h*.^[^ ^152^, ^154^, ^155^ ^]^ The thickness variation is related to the modulus of the wood sponge. A low‐modulus wood sponge is more easily compressed by a small force and has higher sensitivity. A high‐modulus wood sponge has a larger working range. Hu et al. constructed a gradient pore structure film by imitating the structure of the skin (Figure 4f).^[^ ^147^ ^]^ TWPSS can mimic this structure by combining wood sponges with different porosities to achieve both high sensitivity and a wide working range.

#### 3.1.2 Previous Research on Piezoresistive Sensors (Aerogel)

**Figure 5:**
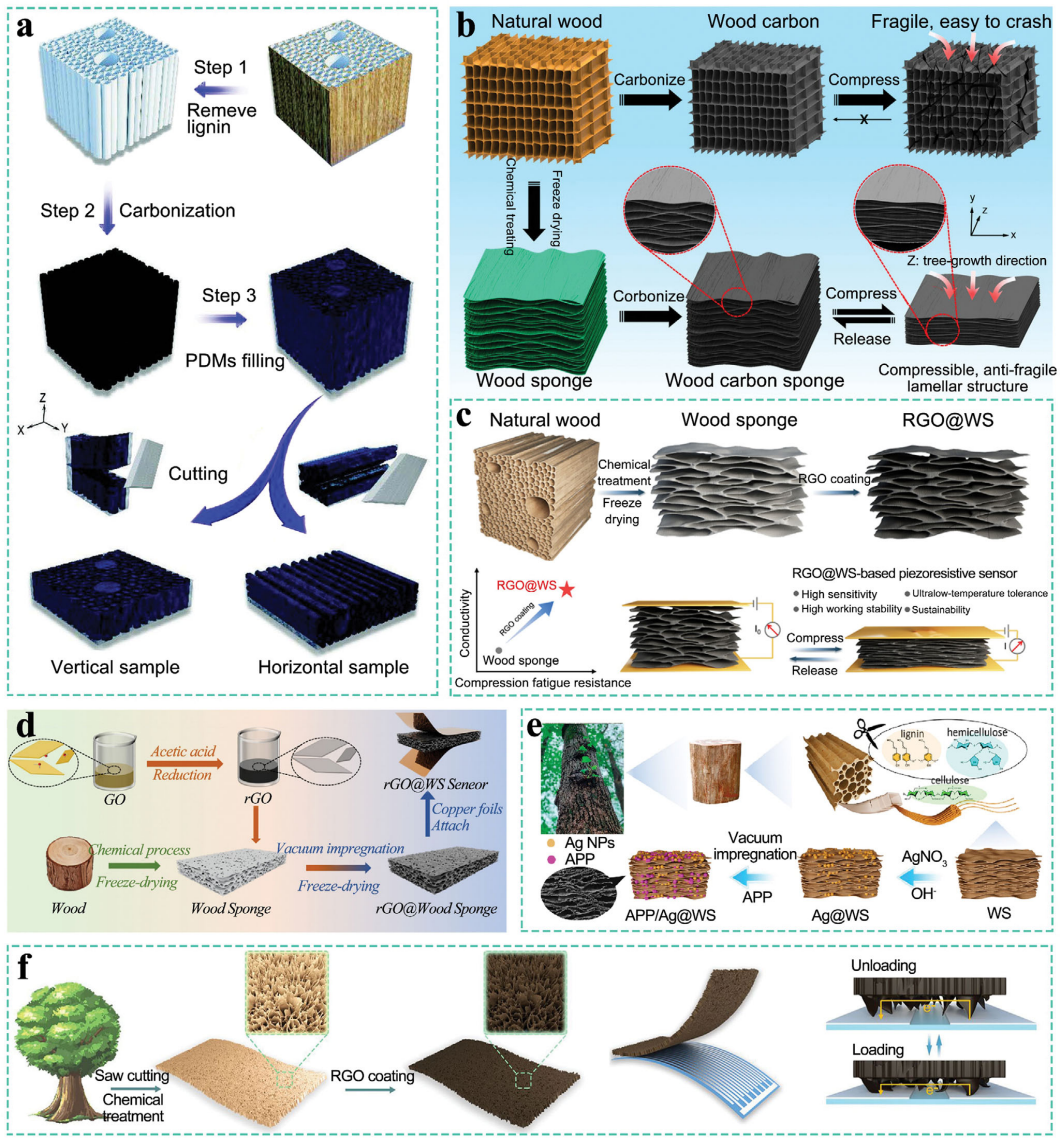
a) Piezoresistive aerogel sensors prepared in different cutting directions. Reproduced with permission.^[^ ^109^ ^]^ Copyright 2023, Wiley‐VCH. b) Highly compressible charcoal sponges with a lamellar structure. Reproduced with permission. Reproduced with permission.^[^ ^108^ ^]^ Copyright 2018, Elsevier. c) A reduced graphene oxide‐coated wood sponge (RGO@WS) with a lamellar structure is used for high‐performance piezoresistive sensors. Reproduced with permission.^[^ ^100^ ^]^ Copyright 2021, American Chemical Society. d) A pressure‐temperature dual‐parameter sensor based on a wood sponge and rGO to detect and distinguish pressure and temperature signals in real‐time. Reproduced with permission.^[^ ^104^ ^]^ Copyright 2024, Royal Society of Chemistry. e) A temperature and pressure sensor modified by SWCNTs and *PEDOT:PSS* with conductive and thermoelectric properties. Reproduced with permission.^[^ ^103^ ^]^ Copyright 2023, American Chemical Society. f) Piezoresistive aerogel sensors with a rough surface structure obtained by horizontal cutting. Reproduced with permission.^[^ ^77^ ^]^ Copyright 2020, American Chemical Society.

The wood sponge inherits the natural anisotropic structure of wood, exhibiting significant differences in compression and tensile moduli across different directions. Along the axial direction, its compressibility is restricted by the cell walls. In contrast, in the radial direction, the distinctive structure formed by the layer‐by‐layer stacking of cell walls endows the wood sponge with an exceptionally high compressibility, which generally exceeds 90%. This high radial compressibility, coupled with low modulus, enables the sensors fabricated from the wood sponge cut in the radial direction to possess a higher level of sensitivity (**Figure** **5**a).^[^ ^109^ ^]^ This is attributed to the fact that in the uncompressed state, only a small contact area exists between two adjacent conductive layers. As the applied pressure increases, the deformation of the wood sponge becomes more significant, leading to increased contact between the adjacent conductive layers. Consequently, a greater number of conductive pathways are formed, thereby altering the resistance of the active layer. Based on this mechanism, the carbonized wood sponge piezoresistive sensor, which uses balsa wood as the raw material, exhibits excellent compression performance (usually >90%), high fatigue resistance (at a strain of 50% and withstanding 10 000 compression cycles), and high sensitivity, enabling its application in the recognition of finger movements (Figure 5b).

SEM images reveal that after delignification and carbonization, the internal morphology of the balsa wood transforms from a lattice structure into a wavy layered structure. This structural transformation endows the wood sponge with enhanced damage resistance.^[^ ^108^ ^]^ However, the high brittleness resulting from direct carbonization cannot be entirely eliminated. Uniform coating of the outer layer of the carbonized wood sponge with Thermoplastic Polyurethane (TPU) or PDMS can, to a certain extent, mitigate this issue.^[^ ^105^, ^156^ ^]^

Sensitivity represents one of the pivotal performance metrics for pressure/strain sensors, a parameter particularly significant in capturing subtle human movements such as pulse and throat vibrations induced by speech. This characteristic, when integrated with artificial intelligence technology, can be further applied in fields such as speech recognition and the assessment of human health conditions. By impregnating or coating the wood sponge with conductive materials like reduced graphene oxide (RGO) nanosheets (Figure 5c), carbon nanotubes, or MXene, the sensitivity of the sensor can be remarkably enhanced, enabling it to outperform the majority of polymer‐based sensors.^[^ ^100^, ^104^, ^119^ ^]^ Furthermore, when detecting subtle human motions, to preclude inaccuracies caused by sweat intrusion, the hydrophobicity of the sensor can be enhanced. This can be achieved by encapsulating components or depositing hydrophobic materials, such as PDMS, on the sensor's surface. Such measures effectively impede the ingress of sweat, ensuring that the sensor maintains reliable operational performance and high sensitivity even in humid conditions.^[^ ^106^, ^157^, ^158^ ^]^ With the development of pressure/strain sensors, the research focus has shifted from single‐function sensors to multifunctional and multi‐parameter ones. This encompasses piezoresistive sensors capable of dual sensing of temperature and pressure, as well as piezoresistive sensors integrated with supercapacitor and electromagnetic shielding functions.

Temperature‐pressure dual‐sensing sensors hold substantial practical significance. Pulse and body temperature represent two critically important vital signs in clinical settings. Real‐time and continuous monitoring of these two signals is essential for effective health management. Nevertheless, when detecting these two signals concurrently, the decoupling of signals and the avoidance of crosstalk between them are the foremost concerns that must be addressed. Xue et al. modified the wood sponge with single‐walled carbon nanotubes (SWCNTs) and poly(3,4‐ethylenedioxythiophene)‐polystyrenesulfonate (PEDOT:PSS). Based on the principles of piezoresistance and thermoelectricity, this modified wood sponge can generate and collect independent voltage and current signals under simultaneous pressure‐temperature stimulation. Furthermore, the research team developed a dual‐parameter pressure‐temperature sensor fabricated from wood sponge and RGO. Owing to the excellent thermal insulation properties of the wood sponge and the thermoelectric performance of RGO, the sensor exhibits both excellent temperature‐sensing and pressure‐sensing capabilities.

Moreover, the decoupling of pressure and temperature signals has been successfully achieved (Figure 5d).^[^ ^104^ ^]^ As a temperature sensor, the wood sponge possesses unique advantages. Along the direction perpendicular to the wood growth direction, there are numerous isolated hollow structures, endowing it with excellent thermal insulation properties. This enables the formation of a temperature difference between the upper and lower surfaces, providing ideal conditions for the generation of thermoelectric voltage. Additionally, dimethyl sulfoxide (DMSO) vapor has been proposed to address the issue of the sensor's resistance variation with temperature, aiming to resolve the crosstalk issue.^[^ ^102^ ^]^ Fire represents an even more extreme scenario, imposing higher demands on the heat resistance and flame‐retardant capabilities of sensors. The bulk wood sponge with a laminated structure modified with silver nanoparticles and ammonium polyphosphate (APP) exhibits excellent fire alarm performance (response time of 0.44 s and duration exceeding 750 s) and reliable repeatability. Moreover, this sensor can still maintain its flexible pressure‐sensing ability under high temperature conditions, with its working range remaining almost unaffected (0–7.5 kPa), demonstrating broad application potential in extreme environments (Figure 5e).^[^ ^103^ ^]^

Owing to the hierarchical porous structure and low tortuosity channels of wood, it serves as an outstanding scaffold material for the fabrication of supercapacitor electrodes. On a macroscopic scale, common methods include carbonizing wood and modifying the carbon materials derived from wood. These modifications include heteroatom doping, as well as the preparation of inorganic compound/carbonized wood composites and conductive polymer/carbonized wood composites.^[^ ^159^ ^]^  The supercapacitor sensor fabricated via vacuum‐assisted adsorption of RGO and Li_2_CO_3_ followed by carbonization exhibits high strength, high specific capacitance, high elasticity, and high elastic recovery rate. Even at a high scan rate of 200 mV·s^−1^, it can still achieve a high specific capacitance of 352 F·g^−1^. Moreover, it demonstrates favorable electrochemical stability and excellent sensing performance.^[^ ^85^ ^]^ Similarly, aiming to combine the functions of a supercapacitor and a pressure sensor, an all‐in‐one multifunctional integrated sensing system with a wide working range (0–25 kPa) and a broad linear sensing range (5–50%) has been constructed. This system involves depositing MXene onto the wood sponge of balsa wood. As a result, it can simultaneously detect surface electromyogram and tactile pressure, enabling real‐time closed‐loop control.^[^ ^119^ ^]^

With the advent of the information age, the extensive application of electronic communication technologies has led to a sharp increase in electromagnetic interference. Traditional electromagnetic shielding materials suffer from issues such as brittleness. Consequently, flexible electromagnetic shielding materials have emerged.^[^ ^160^ ^]^ Flexible electromagnetic shielding materials based on wood have been extensively studied.^[^ ^161^, ^162^, ^163^, ^164^ ^]^ Shen et al. fabricated an electromagnetic shielding material and pressure sensor by infiltrating anisotropic wood (PW) aerogels with conductive polypyrrole (PPy) nanoparticles (NPs). When uncompressed, the porous structure of the wood sponge provides multiple reflection interfaces, which serve as effective sites for electromagnetic wave attenuation (shielding effectiveness >20 dB).^[^ ^84^ ^]^ Another strategy involves incorporating carbon nanotubes (CNTs)/MXene composite nanosheets into the wood sponge through vacuum impregnation. Then, a hydrophobic and multifunctional wood‐derived composite is fabricated by coating with PDMS, achieving an electromagnetic shielding performance of 29.3 dB.^[^ ^165^ ^]^

The above‐mentioned piezoresistive aerogel sensors are based on the highly resilient multi‐layer structure of the wood sponge in the radial direction. However, Guan et al. adopted a different strategy. Their study revealed that circular saw cutting generates ribbon‐shaped microstructures with controlled surface roughness on the tangential plane of wood. Upon compression, the contact area between the rough surface and the electrodes increases, thereby reducing the resistance. The sensor also exhibits favorable performance (Figure 5f).^[^ ^77^ ^]^ The conclusions drawn by Guan et al. seemingly conflict with those previously reported by Huang et al.^[^ ^109^ ^]^ However, in fact, there are significant distinctions. Huang et al. first delignified the wood, followed by carbonization, and finally cut the composite material using a self‐made cutting tool. SEM images reveal that the surface roughness in the vertical direction is relatively low. In contrast, Guan et al. initially opted to cut with a circular saw blade, endowing the material with a highly rough surface from the start, and the subsequent processes preserved this characteristic.^[^ ^77^ ^]^ This strategy for modifying the contact area between wood and electrodes can be subsumed under the category of surface microstructure construction, a practice frequently encountered in polymer‐based pressure sensors. It further provides a strategy: through the control of processing techniques and pretreatment methods, the roughness of the wood structure can be regulated, thereby enabling the design of sensor sensitivity and working range.

The core limitation of piezoresistive aerogel sensors stems from inherent trade‐offs between performance parameters and material properties, particularly the conflict between sensitivity and working range. While low‐modulus wood sponges achieve high sensitivity through large deformation, this simultaneously constrains the working range due to rapid compression saturation and compromises linearity. Processing challenges further arise from carbonized materials; although this can be mitigated by coating with TPU or PDMS, long‐term fatigue resistance remains compromised. The sensors also need to address environmental influences: susceptibility to moisture (e.g., sweat) necessitates hydrophobic treatments or encapsulation, while temperature‐induced resistance drift requires mitigation strategies such as DMSO vapor treatment to reduce thermal interference, collectively increasing design complexity.

Agricultural wastes not derived from wood, such as corn stalks, have also been experimentally employed via a top‐down approach to prepare TWPSS aerogels. Such an approach has expanded the raw material sources for bio‐based sensors.^[^ ^166^ ^]^ The raw materials, manufacturing processes, and properties of piezoresistive aerogel TWPSS are summarized in **Table** **3**.

**Table 3:** Raw materials, fabrication processes, and performance of piezoresistive aerogel TWPSS.

Main material	Preparation method	Size: thickness × length × width [mm]	Work range [kPa]	Sensitivity: Gauge factor [kPa^−1^] or ∆R/R	Stability [cycle tests]	Response time, Recovery time [ms, ms]	Refs.
Balsa wood, RGO	delignification, immersion, reduction	0.8 × 10 × 10	<60	1.85	10 000	150, 100	[77]
Balsa wood	delignification, freeze drying, carbonization	1 × 10 × 5	≈ 70	–	10 000	–	[108]
Balsa wood, PDMS	delignification, freeze drying, immersion	0.5 × 3 × 3	≈ 100	10.74	13 000	20, ‐	[109]
Balsa wood, RGO	delignification, freeze drying, immersion, reduction, immersion	20 × 20 × 20	≈ 12	0.32	10 000	120, 60	[100]
Balsa wood, RGO, PDMS	delignification, freeze drying, immersion, reduction, immersion	10 × 10 × 10	≈ 50	4.93 (0–5 kPa), 0.75 (5–50 kPa)	1000	160, 200	[106]
Balsa wood, Flake graphite	delignification, freeze drying, immersion, freeze drying, carbonization	3 × 40 × 10	–	–	5000	–	[85]
Balsa wood, MXene, PVA	delignification, freeze drying, immersion, directional freezing, freeze drying	10 × 10 × 10	≈ 25	1.31 (∆R/R)	5000	–	[119]
Balsa wood, TPU	delignification, carbonization	15 × 15 × 12	≈ 100	76.18 (0–1 kPa), 20.95 (1–10 kPa), 2.25 (10–100 kPa)	10 000	–	[105]
Balsa wood, FeCl_3_, Pyrrole	delignification, freeze drying, immersion	25 × 25 × 25	–	1.69–2.30	–	–	[84]
Balsa wood, PEDOT:PSS,	delignification, freeze drying, immersion	10 × 10 × 10	–	67.2 (0–1 kPa), 5.12 (1–5 kPa), 0.30 (5–20 kPa)	2000	108, ‐	[102]
Balsa wood, PEDOT:PSS, SWCNTs	delignification, freeze drying, immersion, freeze drying	10 × 10 × 10	≈ 20	1.05 (0–10 kPa), 0.35 (10–20 kPa)	500	20, ‐	[103]
Balsa wood, RGO	delignification, freeze drying, immersion	15 × 15 × 15	0.2–100	1.18 (0–10 kPa), 0.43 (10–100 kPa)	1000	110, 120	[104]
Orn stover pith, PEDOT:PSS, CNTs	delignification, freeze drying, immersion, heat drying	10 × 10 × 20	5.5–500	10.30 (0–6.5 kPa), 1.80 (6.5–5 kPa), 0.50 (5–20 kPa)	–	<400	[166]
Wood, AgNO_3_, APP	delignification, freeze drying, impregnated by vacuum	10 × 10 × 40	≈ 7.5	Before fire 103.55 (0–0.5 kPa), 40.50 (0.5–4 kPa), 0.69 (4–7.5 kPa); After fire 226.03 (0–0.3 kPa), 1.42 (0.5–7.5 kPa)	Before fire 3000, After fire 3000	20, ‐	[111]

Note: “‐” means not mentioned in the references. Pressure range is standardized to kPa, the sensitivity is standardized to kPa^−1^ for consistency. Sensitivity is presented as either Gauge Factor (GF = (∆R/R₀)/∆P, ∆P in kPa) or (∆R/R₀). Data without explicit ∆P values are reported as originally published.

#### 3.1.3 Principle of Piezoresistive Sensor (Hydrogel)

The physical piezoresistive effect primarily arises from changes in the intrinsic resistivity of the active layer under mechanical deformation. In most hydrogel‐based piezoresistive TWPSS, a delignified wood sponge serves as the structural framework, which is typically integrated with polymeric matrices such as poly(vinyl alcohol) (PVA), N, N’‐methylenebisacrylamide (MBA), or polydimethylsiloxane (PDMS). The sensing capability is derived from a conductive network constructed through the intrinsic ion channels of the wood and/or conductive fillers incorporated within the hydrogel matrix. In systems where ion transport dominates, mechanical compression, particularly in the radial direction, triggers bending of the cellulose microfibers, reducing interfibrillar spacing and constricting the original ion‐conducting channels (Figure 4b). This deformation facilitates faster ion mobility, thereby decreasing the resistance of the hydrogel sensor. When the hydrogel is compressed or bent perpendicular to the fiber direction, the channels within the wood are blocked by the cell walls, resulting in a lack of pathways for ion transport. Consequently, the electrical conductivity in this direction is poor. Moreover, the deformation in this direction is primarily characterized by changes in the distance between cellulose fibers and wood lumens, which is not significant. This leads to only a minor change in electrical resistance, making it difficult to meet the performance requirements of sensors.^[^ ^87^ ^]^  This is the reason why the electrodes of the piezoresistive hydrogel TWPSS are installed on both sides along the fiber direction. In contrast, for aerogels, the electrodes are installed on both sides perpendicular to the fiber direction.

For hydrogel composites incorporating conductive fillers, the sensing behavior is determined by both percolation theory and the tunneling effect. The percolation theory can be described as follows: when the content of conductive fillers is low, the conductive fillers are separated from each other, and the resistivity of the active layer is determined by that of the hydrogel. When the content of conductive fillers reaches a certain threshold, the conductive fillers connect with each other to form a conductive path, and the resistivity of the hydrogel drops sharply as the filler content increases. When the content of conductive fillers is in the percolation region, the deformation caused by the compression of the composite material will alter the distance between the conductive fillers, thereby changing the conductive network in the composite material and its resistivity (Figure 4d). This is dependent on the volume content of conductive fillers in the active layer. When there is no external force, the polymer composite is only partially conductive. When an external pressure is applied, the average distance between the conductive particles decreases and breaks through the threshold, and the conductive path changes accordingly, and the resistivity of the hydrogel decreases.^[^ ^167^, ^168^, ^169^ ^]^ The tunneling effect means that in an insulating polymer matrix, a conductive network can be formed not only through the contact between conductive materials but also through adjacent conductive materials within a certain distance, allowing electrons to form a quantum tunnel through a thin polymer layer. The tunneling effect occurs due to the probability of electrons transitioning from a low‐energy state to a high‐energy quantum state, leading to the formation of a tunneling current.^[^ ^170^, ^171^, ^172^ ^]^ Hydrogel piezoresistive TWPSS are typically not driven by a single mechanism but operate through the collaborative action of the above mechanisms.

#### 3.1.4 Previous Research on Piezoresistive Sensors (Hydrogel)

**Figure 6:**
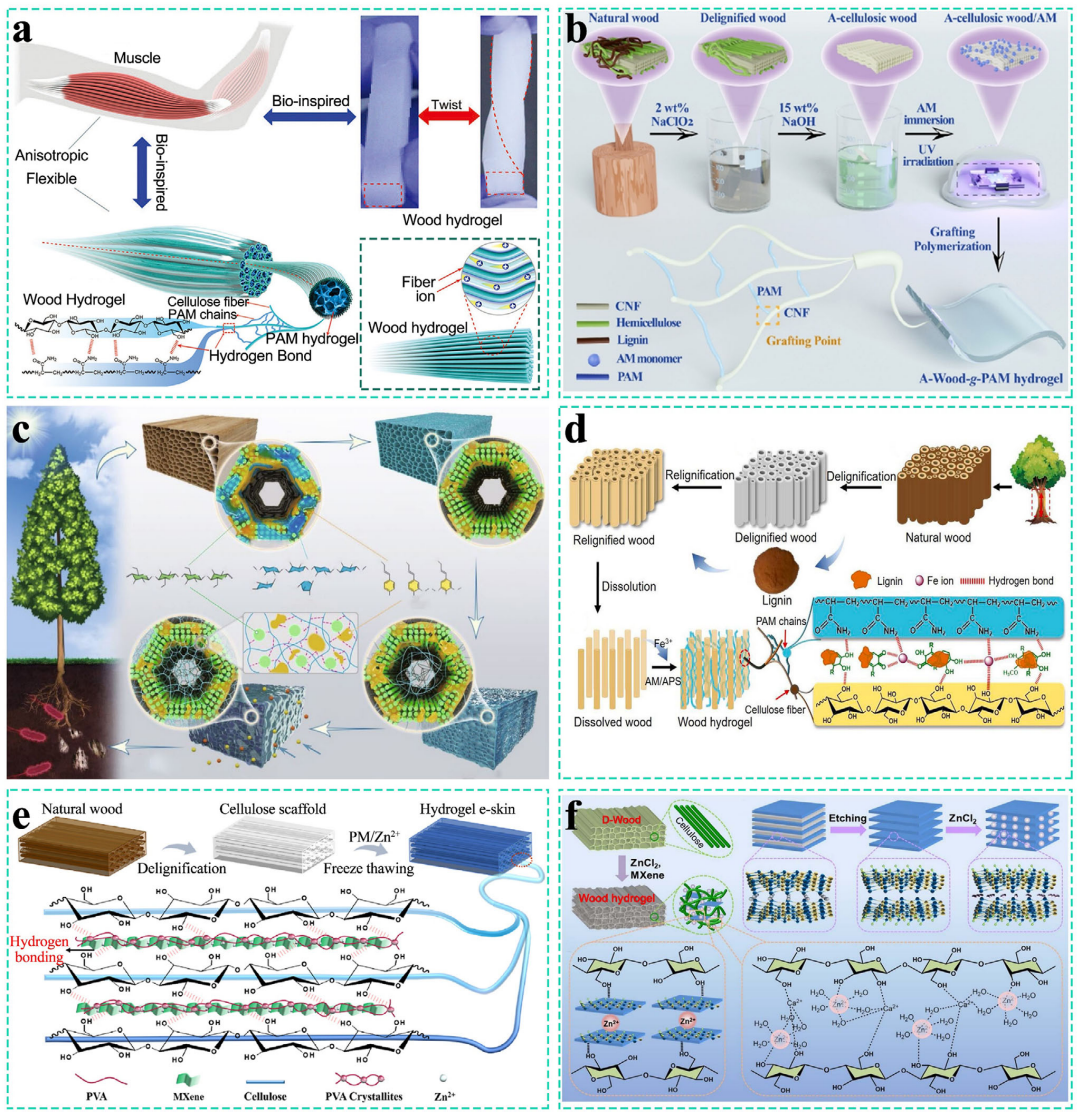
a) A highly anisotropic, strong, and electrically conductive wood‐based hydrogel inspired by muscle. Reproduced with permission.^[^ ^86^ ^]^ Copyright 2018, Wiley‐VCH. b) A hydrogel with high tensile properties prepared via ultraviolet grafting. Reproduced with permission.^[^ ^99^ ^]^ Copyright 2021, American Chemical Society. c) An all‐wood hydrogel constructed by cross‐linking cellulose fibers, PVA chains, and lignin molecules through the Hofmeister effect. Reproduced with permission.^[^ ^95^ ^]^ Copyright 2022, Springer Nature. d) An all‐wood tough hydrogel assembled with anisotropic mechanical properties through the formation of dynamic bonds among cellulose, natural lignin, PAM chains, and iron ions. Reproduced with permission.^[^ ^116^ ^]^ Copyright 2021, Elsevier. e) A freeze resistant and robust wood derived hydrogel using MXenes as fillers, applicable to high performance electronic skins and wearable sensor devices. Reproduced with permission.^[^ ^101^ ^]^ Copyright 2022, Elsevier. f) A wood‐based hydrogel with good anisotropy, excellent mechanical properties, high electrical conductivity, and extreme temperature resistance, prepared using MXenes as fillers under the action of Zn^2^⁺ ions. Reproduced with permission.^[^ ^88^ ^]^ Copyright 2023, Royal Society of Chemistry.

Cellulose‐based hydrogels have emerged as a promising material for high‐performance wearable electronics, owing to their intrinsic biocompatibility, flexibility, and biodegradability.^[^ ^173^, ^174^ ^]^ Wood's natural channel‐like structure and its negatively charged surface endow them with the ability to selectively transport ions. This unique property renders wood a potential candidate for sensor materials.^[^ ^175^ ^]^ In the hydrogel composed of wood sponge and polymer, the wood sponge is a tightly cross‐linked and well‐extended network. The highly ordered arrangement within it endows the wood sponge with excellent tensile strength along the fiber direction and good compressive resilience in the perpendicular direction. This effectively addresses the issue of weak mechanical properties of traditional hydrogels (**Figure** **6**a).^[^ ^86^ ^]^ In the hydrogel, the polymer features a sparse cross‐linking structure. It binds to the wood sponge through covalent or non‐covalent bonds, which further ensures the mechanical strength of the hydrogel.^[^ ^157^, ^176^, ^177^ ^]^ Furthermore, as a rigid supporting material, the wood sponge can effectively mitigate the problem of excessive water absorption in the hydrogel, thereby enhancing its stability in an aqueous environment.^[^ ^78^ ^]^

Based on the strategy of utilizing the inherent ion channels of wood sponge for sensing, hydrogel TWPSS fabricated from various polymers and wood sponge has been developed. For instance, polyacrylamide (PAM) has been employed for in situ compounding with balsa wood sponge. The resultant hydrogel exhibits enhanced flexibility. Meanwhile, a robust interface is formed between the cellulose and PAM chains, which elevates its tensile strength to (16.47 ± 1.40 MPa).^[^ ^97^ ^]^ Building upon this, a green and rapid UV‐grafting synergistic strategy was developed. This approach enabled the grafting of PAM onto the wood sponge without the requirement of an initiator, thereby further augmenting the tensile performance of the hydrogel to 30.76 MPa (Figure 6b).^[^ ^99^ ^]^ Similarly, without modifying the wood sponge or employing chemical cross‐linkers, Yan et al. synthesized an all‐wood hydrogel by leveraging the simple Hofmeister effect. This hydrogel features good flexibility, electrical conductivity, and adjustable mechanical strength, with its tensile strength reaching 36.5 MPa. This is due to the introduction of lignin molecules and PVA, as strong hydrogen bonds, physical entanglements, and van der Waals forces are formed between them and the cellulose nanofibers. The hydrogel's electrical conductivity and sensitivity endow it with the capacity to detect minute human motions (Figure 6c).^[^ ^95^ ^]^ Inspired by the chemical principles of catechols, this team formulated an all‐wood tough hydrogel composed of wood sponge, lignin, PAM, and Fe^3^⁺. In the presence of ammonium persulfate (APS), Fe^3^⁺ and the catechol groups in lignin trigger rapid self‐gelation of the hydrogel, resulting in the formation of reversible hydrogen bonds and Fe^3^⁺‐catechol metal coordination bonds. These interactions endow the sensor with enhanced flexibility and tensile performance (with a strain range of up to 50%) (Figure 6d).^[^ ^116^ ^]^ In another study, Fe^3^⁺ ions were introduced into carboxyl‐grafted delignified wood impregnated with acrylamide (AM) and acrylic acid (AA). Through in situ polymerization, a hydrogel TWPSS was formed. Under dynamic covalent crosslinking, both the mechanical properties were improved and the electrical conductivity was enhanced.^[^ ^87^ ^]^ Furthermore, a self‐healing hydrogel pressure sensor was developed to function as an electronic skin. The highlight of this electronic skin lies in its ability, while functioning as a sensor, to endure complex deformations such as bending and stretching, and to self‐repair after fracture.^[^ ^96^ ^]^

The above research is based on the ion‐channel‐based sensing characteristics of cellulose itself. The wood sponge hydrogel, with its compressibility and flexibility, can effectively preserve its internal channels when subjected to external forces or undergoing deformation. This preservation of internal channels provides a prerequisite for ion transport.^[^ ^178^, ^179^ ^]^ Specifically, under external forces, the original conduits within the wood are compressed, and when the hydrogel bends along the fiber direction, the distance between cellulose fibers decreases. These structural changes accelerate ion transport, leading to a corresponding change in electrical resistance.

Another approach entails embedding conductive fillers (such as carbon nanotubes, MXene, and RGO) into the hydrogel. The resistance variation is attributed to contact‐effect and tunneling‐effect mechanisms, while the overall conductivity arises from electron migration within the filler network. Based on this mechanism, a variety of TWPSS have been developed. For instance, a hydrogel with high toughness and good electrical conductivity was prepared by coupling a wood sponge with a polyvinyl alcohol (PVA)/MXene nanosheet network through a simple freeze‐thaw process.^[^ ^88^ ^]^ On this basis, the addition of ZnCl_2_ serves to promote the exfoliation and dispersion of MXene while inducing the rearrangement of cellulose fibers. An extensive hydrogen bond network is constructed between cellulose and MXenes. The ionic coordination between Zn^2^⁺ and hydroxyl groups provides a secondary cross‐linking network for the composite hydrogel. The prepared wood‐based hydrogel exhibits good anisotropy, mechanical properties, electrical conductivity, and extreme temperature resistance (Figure 6e).^[^ ^101^ ^]^

In terms of key performance, the factors influencing the sensitivity of aerogel and hydrogel TWPSS differ, which is related to their sensing mechanisms. For piezoresistive aerogel TWPSS, owing to the low modulus of the wood sponge, large‐scale deformation can occur under relatively small pressures. This leads to a rapid increase in conductive pathways, thereby enhancing the sensitivity of the sensor. If the internal porosity of the wood is altered to further regulate the compression modulus of the wood sponge, the adjustment of the sensor's sensitivity can be achieved. However, solely reducing the compression modulus is detrimental to other performance indicators, such as the working range and linearity. A low modulus will cause the wood sponge to quickly reach its compression limit, thereby reducing its working range. Moreover, it is difficult for the sensor to maintain good linearity. As the applied force increases and the wood sponge is compressed, its modulus rises, and the increment of conductive pathways decreases. This is the reason why the sensitivity of the sensor is limited within a certain range in different stages.

For piezoresistive hydrogel TWPSS, the sensitivity is related to the type, content, dispersion, and morphology of the fillers. When the filler concentration approaches the electroosmotic threshold, the sensor typically exhibits the maximum resistance change (Figure 6f).^[^ ^88^ ^]^ All of this is premised on the uniform distribution of fillers. Fillers distributed in an island‐like pattern are unable to construct a continuous conductive network within the hydrogel, rendering the relative resistance change (ΔR/R) insensitive to external stimuli. It should be noted that, generally, the key to enhancing sensitivity based on the tunneling effect lies in increasing the tunneling resistance or the proportion of tunneling resistance relative to the total resistance, rather than the total resistance itself.^[^ ^180^, ^181^ ^]^ Likewise, a balance needs to be struck between excellent sensitivity and high linearity. Moreover, sensitivity can also be enhanced by constructing microstructures at the interface between the material and the electrode.

For hydrogel TWPSS, especially those relying on the conduction of conductive fillers, potential friction results in a longer recovery time during the release cycle.^[^ ^154^, ^182^, ^183^ ^]^ Significant hysteresis leads to irreversible sensing characteristics of hydrogel TWPSS under dynamic loading conditions.^[^ ^184^, ^185^ ^]^ This is because the weak bonding between fillers and the polymer matrix allows fillers to easily slide within the matrix under large‐scale stretching, meaning they take a long time to return to their original positions after strain release.^[^ ^180^, ^186^ ^]^ In contrast, piezoresistive aerogels contain numerous air‐filled chambers within the wood sponge, which mitigates viscoelasticity and enhances response speed.

Tensile performance is another crucial parameter determining the working range of a sensor.^[^ ^187^, ^188^ ^]^ This is precisely why the compressible amount and the corresponding pressure at maximum compression are regarded as important indicators for aerogels.^[^ ^183^, ^189^ ^]^ Natural wood exhibits a tensile strain range of only 0–5%. In contrast, hydrogels prepared by in situ chemical polymerization of PAM in wood sponges achieve a tensile elongation of 15.99 ± 1.81%.^[^ ^97^ ^]^ Yan et al. synthesized a composite using cellulose as the rigid framework via the simple Hofmeister effect, with an axial strain of 438% (noting that this represents the ultimate tensile limit of the hydrogel, rather than its working range).^[^ ^95^ ^]^

The limitations of piezoresistive hydrogel sensors focus on dynamic response and structural dependence. Significant hysteresis occurs due to weak bonding between conductive fillers and the polymer matrix, leading to prolonged recovery times under dynamic loading and directly impairing real‐time response accuracy.^[^ ^190^ ^]^ Although wood‐based sponges have improved tensile strength (reaching up to 36.5 MPa in some cases) and significantly enhanced strain compared to natural wood, their tensile strain range remains limited (mostly <50%), which restricts their application in large‐deformation scenarios. Simultaneously, long‐term immersion induces swelling; while wood sponges can constrain water absorption, cross‐linking structures still require optimization to improve durability. The raw materials, manufacturing processes, and properties of piezoresistive hydrogel TWPSS are summarized in **Table** **4**.

**Table 4:** Raw materials, fabrication processes, and performance of piezoresistive hydrogel TWPSS.

Main material	Preparation method	Size: thickness × length × width [mm]	Elongation	Sensitivity gauge factor [kPa^−1^] or ∆R/R	Tensile strength [MPa]		Stability [cycle tests]	Refs.
					Parallel	Across		
Balsa wood, Ti_3_AlC_2_, ZnCl_2_, CaCl_2_, LiF, PDMS	delignification, freeze drying, vacuum immersion	1 × 30 × 10	–	0.0497(0–20 kPa), 0.0142 (20–520 kPa), 0.04(400 kPa)	0.8	–	100	[88]
Balsa wood, AM, MBA, APS	delignification, freeze drying, vacuum immersion	1 × 25 × 5	1.32% tensile strain	0.0021 (0.1–25 kPa), 0.00037 (25–125 kPa), 0.00013 (125–245 kPa), 0.00004 (245–490 kPa)	19.8	2.7	300	[78]
Balsa wood, AM, FeCl_3_·6H_2_O, AA, MBA	delignification, freeze drying, vacuum immersion, freeze drying	1 × 10 × 50	69.1% compressive strain	0.0615 (0.02 kPa),0.047 (0.2 kPa),0.04 (0.4 kPa)	8.2	–	100	[87]
Balsa wood, AM, MBA	delignification, immersion	1 × 30 × 30	15.99 ± 1.81% tensile strain	–	16.47 ± 1.40	–	100	[97]
Balsa wood, AM, MBA	delignification, immersion, ultraviolet grafting	1 × 30 × 30	16.07% tensile strain	–	30.76	1.59 ± 0.03	50	[99]
Balsa wood, vinyl alcohol	delignification, immersion	1 × 20 × 60	438% tensile strain	3.21 (0–108% strain) (∆R/R), 6.16 (108–180% strain) (∆R/R)	36.5	2.6	–	[95]
Balsa wood, AM, APS, FeCl_3,_ LiCl, DMAc	delignification, immersion	15 × 15 × 20	up to ≈ 50% tensile strain	0.0031 (0.1–60 kPa), 0.0022 (60–235 kPa), 0.0006 (>235 kPa)	–	–	200	[116]
Balsa wood, PVA	delignification, vacuum impregnation	2 × 20 × 60	463% tensile strain	4.6 (∆R/R)	21.2	4.3	–	[96]
Balsa wood, AM, AA, FeC_l3_	delignification, immersion, UV, submerged	10 × 40 × 40	–	–	42.24	4.24	45	[191]
Balsa wood, 1‐allyl‐trimethylimidazole chloride salt, 1‐butyl‐trimethylimidazole chloride salt, AA, MBA, FeCl_3_	delignification, immersion (ionic liquid), vacuum drying, immersion, UV	1 × 10 × 50	50% tensile strain	8.6 (≈35 % strain) (∆R/R)	9.0	0.97	>500 (train of 25 %, 33 Hz)	[192]

Note: “‐” means not mentioned in the references. Pressure range is standardized to kPa, the sensitivity is standardized to kPa^−1^, strength is standardized to MPa for consistency. Sensitivity is presented as either Gauge Factor (GF = (∆R/R₀)/∆P, ∆P in kPa) or (∆R/R₀). Data without explicit ∆P values are reported as originally published. “Parallel” and “Across” means parallel and across to the direction of wood growth.

### 3.2 Capacitive Sensor

#### 3.2.1 Principle of Capacitive Sensor

The basic structure of a capacitive sensor consists of an elastic insulating medium placed between two parallel conductive plates. Its working principle is to measure the capacitance change of the parallel plate capacitor under pressure or strain.^[^ ^193^ ^]^ Generally, the capacitance of a planar parallel plate capacitor can be expressed by Equation (2):

(2) $$C=k\cdot \varepsilon_{0}\frac{l\cdot w}{d}$$

where *k* is the relative permittivity of the polymer dielectric, ε_0_(8.85 × 10^−12^ F·m^−1^) is the permittivity of vacuum, *l* and *w* are the length and width of the parallel electrodes respectively, and *d* is the distance between the two parallel electrodes.

**Figure 7:**
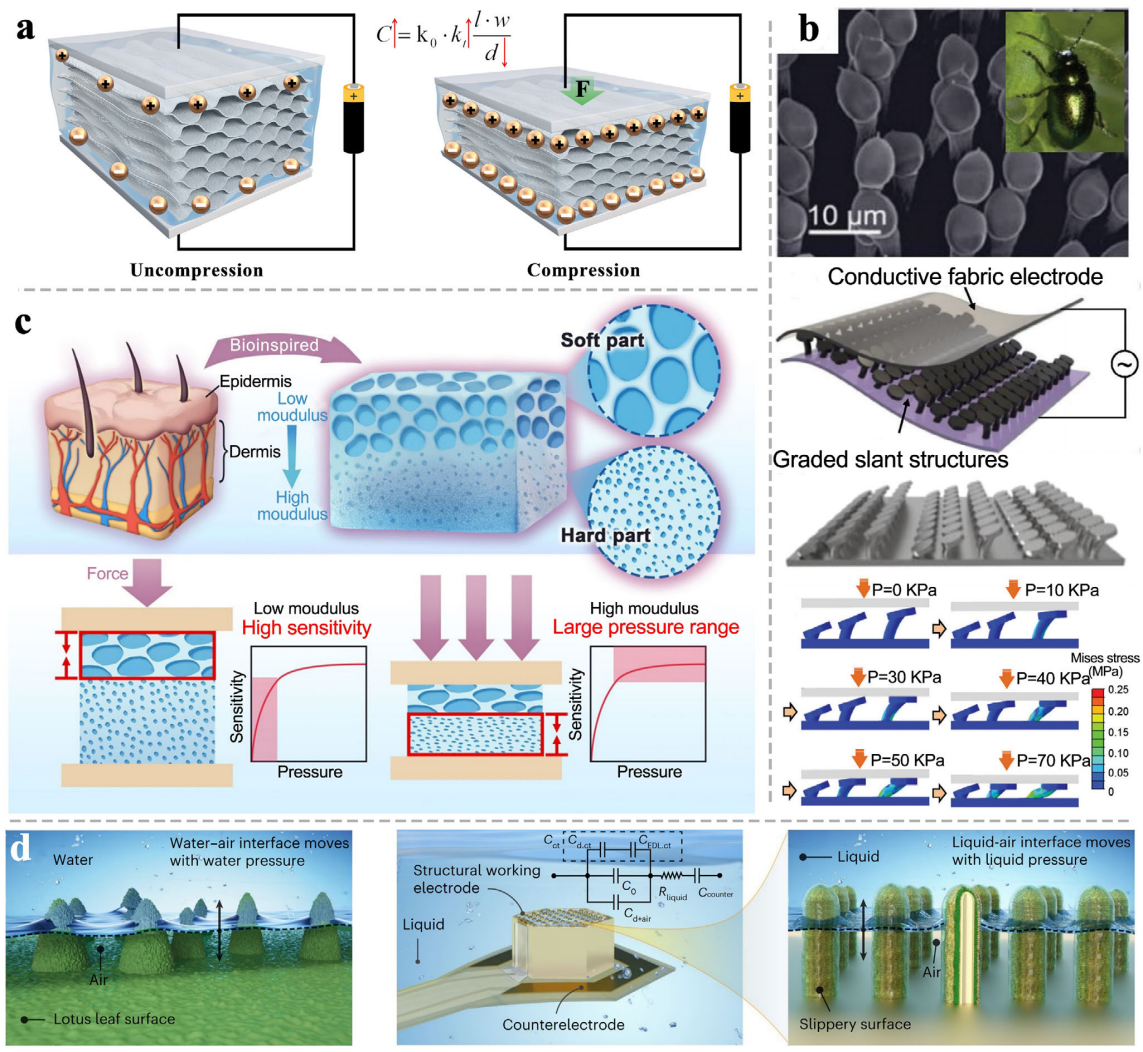
a) Sensing principles of capacitive TWPSS. b) A novel dielectric layer based on beetle‐inspired gradient slant structures enables the capacitive pressure sensor with extensive linearity range. Reproduced with permission.^[^ ^196^ ^]^ Copyright 2024, Wiley‐VCH. c) Cellulose hydrogel with a layered porous structure composed of a low‐modulus soft layer and a high‐modulus hard layer. Reproduced with permission.^[^ ^197^ ^]^ Copyright 2025, Springer Nature. d) a pressure sensor that uses the solid–liquid–liquid–gas multiphasic interfaces and the trapped elastic air layer to modulate capacitance changes with pressure at the interfaces. Reproduced with permission.^[^ ^198^ ^]^ Copyright 2023, Springer Nature.

It can be found that the change in capacitance will vary with the change in the vertical distance between the two electrodes, and the permittivity of the material between the electrodes will also affect the magnitude of the capacitance (**Figure** **7**a).^[^ ^194^, ^195^ ^]^ Assuming that there is a linear elastic dielectric material between the two plates, the initial sensitivity of the capacitive pressure sensor can be deduced as Equation (3):

(3)) $$S=\frac{d\left(\frac{\Delta c}{c_{0}}\right)}{dP}=\frac{1}{k_{0}}\left(\frac{k}{E}+\frac{\partial k}{\partial P}\right)$$

where *P* is the applied pressure, and *E* is the compression modulus of the dielectric material. This sensitivity equation holds only when the applied pressure is much smaller than the modulus of the dielectric material, that is, P≪E.

As indicated by the formula, the optimization of capacitive sensor performance can be realized through two approaches: 1) employing low‐modulus materials and 2) modulating the effective permittivity of the pressure‐sensitive dielectric.^[^ ^199^ ^]^ Introducing air gaps within the dielectric or at the electrode‐dielectric interface simultaneously fulfills both requirements.^[^ ^200^ ^]^ Notably, wood sponge uniquely satisfies these criteria. As a superior dielectric material, cellulose enhances permittivity, while the wood sponge's low elastic modulus and high porosity synergistically optimize its mechanical and electrical responses. In the initial state, the actual permittivity of the dielectric layer is jointly determined by cellulose and air. During compression, the voids in the wood sponge gradually decrease, leading to an increase in the actual permittivity of the dielectric layer. This expands the sensor's detection range but compromises its linearity—specifically, high sensitivity cannot be maintained under high pressure. This is because compression rapidly reduces the air gaps until the wood sponge itself is subjected to pressure, causing a sudden increase in modulus. Inkyu Park et al. proposed an interesting scheme: beetle‐inspired gradient slant structures designed to enhance capacitive pressure sensors (Figure 7b).^[^ ^196^ ^]^ Similarly, wood could achieve similar structures through machining methods such as sawing to improve sensitivity and sensing range.

In addition, wood sponge hydrogel is also a potential solution. Water molecules exhibit high polarizability and relatively high permittivity, which can enhance the sensitivity of capacitive sensors while ensuring electron insulation.^[^ ^201^ ^]^ Lu et al. designed a cellulose hydrogel capacitive sensor consisting of a soft layer with large pores and a hard layer with small micropores. The macropores in the soft layer facilitate significant deformation and charge accumulation, endowing the sensor with exceptional sensitivity to low pressures (Figure 7c).^[^ ^197^ ^]^ A potential approach—combining wood sponges with different moduli—might achieve similar sensing performance, though this strategy would come at the expense of linearity. In another study, the solid‐liquid‐gas multiphase interface and the trapped elastic air layer were utilized to modulate pressure‐dependent capacitance variations at the interface (Figure 7d).^[^ ^198^ ^]^ Currently, wood‐derived capacitive pressure sensors focus more on material design itself, whereas structural designs such as those mentioned above could represent another effective optimization direction.

#### 3.2.2 Previous Research on Capacitive Sensors

**Figure 8:**
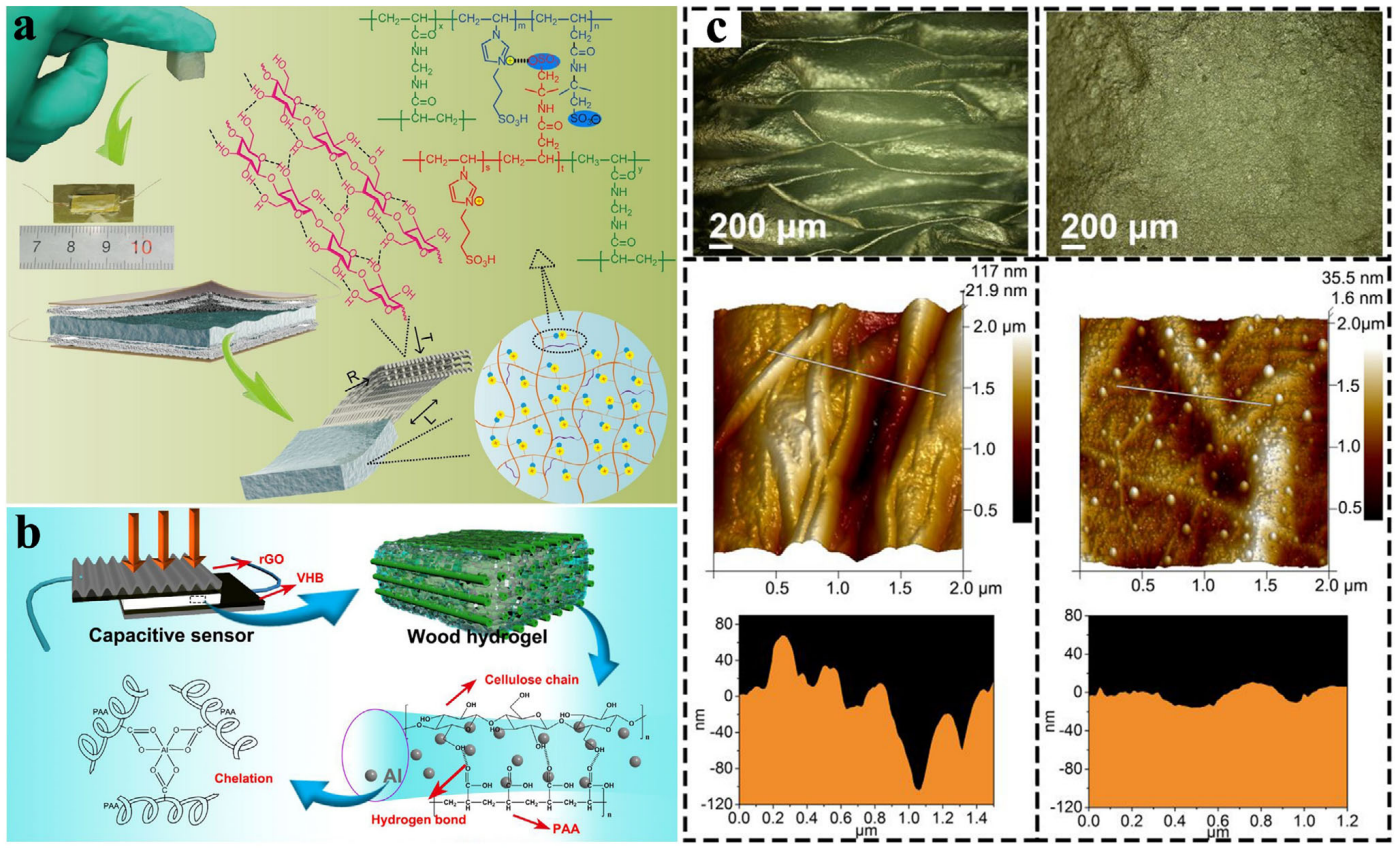
a) The dielectric layer material of the capacitive sensor prepared by introducing polyacrylic acid (PAA) into the wood sponge. Reproduced with permission.^[^ ^79^ ^]^ Copyright 2020, Royal Society of Chemistry. b) A tactile sensor with an electric double layer capacitance structure formed by delignification, impregnation, and gelation to create a rigid wood aerogel/poly (ionic liquid) (WA/PIL) hydrogel. Reproduced with permission.^[^ ^118^ ^]^ Copyright 2020, American Chemical Society. c) Wrinkled RGO film. Reproduced with permission.^[^ ^79^ ^]^ Copyright 2020, Royal Society of Chemistry.

Currently, research on capacitive TWPSS remains relatively limited, with a primary focus on hydrogels. This is attributed to the high polarizability and relatively large dielectric constant of water molecules; enhancing the dielectric properties of the dielectric layer in capacitive sensors can effectively improve their performance. Sun et al. developed a hydrogel that inherits the excellent mechanical, insulating, and dielectric properties of wood, particularly under wet conditions (**Figure** **8**a). This hydrogel exhibits advantages such as non‐flammability and high ionic conductivity. When used as the dielectric layer with reduced RGO as the electrode, the fabricated sensor demonstrates a wide working range and a response time of less than 0.25 s.^[^ ^79^ ^]^

Building on this work, the same team prepared a wood‐based hydrogel with favorable mechanical properties by incorporating polyacrylic acid (PAA) into the wood sponge (Figure 8b). Using this hydrogel as the dielectric layer and reduced RGO as the electrode, the sensor achieved gradient pressure sensitivities (58.4, 41.8, and 27.0 MPa^−1^) within a pressure range of ≈1.6 MPa. This capacitive TWPSS can stably monitor human activities under both large impacts (1.3 MPa) and small general stresses (0.1 MPa).^[^ ^118^ ^]^ The team further selected wrinkled RGO as the electrode material; its wrinkles significantly enhance the capacitance response to pressure, representing a strategy for constructing surface microstructures (Figure 8c).^[^ ^195^ ^]^ Another approach for capacitive TWPSS involves using silver wires and flexible transparent wood as electrodes, with a PDMS film featuring a pyramid‐like structure as the stimulus‐responsive layer.^[^ ^89^ ^]^

More attempts on capacitive pressure/strain sensors have focused on fabricating cross‐digital capacitive touch sensors on the original wood veneer through methods such as laser‐induced graphitization,^[^ ^202^, ^203^ ^]^ printing,^[^ ^204^ ^]^ coating with conductive inks,^[^ ^205^ ^]^ and scribing with a pencil.^[^ ^206^ ^]^ However, these studies are not within the scope of discussion in this paper.

Regarding sensitivity, an increase can be attained by decreasing the modulus of the dielectric material and modifying its dielectric constant.^[^ ^207^ ^]^ Introducing small air gaps within the dielectric layer represents one of the most commonly adopted strategies.^[^ ^195^ ^]^ The dielectric layer of TWPSS consists of wood sponge and the materials filling its voids. Sensitivity can be enhanced by constructing microstructures using the cavities in the wood. Additionally, doping or coating the wood sponge with high‐κ dielectric materials will further increase the dielectrostrictive effect.^[^ ^199^, ^208^ ^]^ However, similarly, this strategy is only effective within the low‐pressure range, and at the cost of deteriorated linearity. This is because as the electrode distance changes, the dielectric constant of the dielectric layer composed of cellulose and air gradually increases. The simultaneous variation of these two variables gives rise to the nonlinear change in capacitance.^[^ ^154^ ^]^

A hybrid sensing strategy integrating piezoresistive and capacitive mechanisms is developed. The dielectric layer consists of a porous nanocomposite material with minimal conductivity and an ultra‐thin electrically insulating layer. The insulating layer exhibits high stiffness and is thus considered non‐deformable, which helps preserve the capacitive component of the hybrid sensor. When the porous nanocomposite demonstrates high electrical conductivity, the capacitive response is weakened, causing the material to function as a purely piezoresistive sensor. Conversely, when the nanocomposite is highly insulating, the piezoresistive response is negligible, and it behaves as a conventional porous medium dominated by capacitive sensing. This could potentially be a promising solution for TWPSS. However, this approach still lags significantly behind commercially available materials such as nickel foam.^[^ ^200^ ^]^

The nonlinear output of capacitive sensors is inherent: during compression, reduced air gaps alter the dielectric constant, which, combined with changes in electrode spacing, causes nonlinear capacitance fluctuations. High sensitivity primarily relies on low modulus and air gaps; however, under high pressure, the abrupt increase in the wood sponge's modulus leads to a sharp drop in sensitivity, restricting effective sensitivity to low‐pressure ranges. Additionally, capacitive sensors are susceptible to environmental influences. Variations in temperature and humidity alter the material's permittivity, resulting in capacitance drift. Nearby electromagnetic equipment (e.g., electric motors, high‐frequency signal sources) also generates stray electric fields that perturb the electric field distribution between the measuring electrodes, causing fluctuations in the measured capacitance value. The raw materials, manufacturing processes, and properties of capacitive TWPSS are summarized in **Table** **5**.

**Table 5:** Raw materials, fabrication processes, and performance of capacitive TWPSS.

Main material	Preparation method	Size: thickness × length × width [mm]	Work range [kPa]	Sensitivity: Gauge Factor [kPa^−1^]	Tensile strength parallel to grain [MPa]	Compressive strength, maximum strain [MPa]	Stability [cycle tests]	Refs.
Poplar, An acrylic elastomer, 1vinylimidazole, 2‐acrylamido‐2methylpropanesulphonic acid, MBA,	Delignification, freeze drying, vacuum immersion	10 × 10 × 10	1200 (70% strain)	0.00967 (<92 kPa), 0.00033 (<1000 kPa)	1.42	1.33, 4.5% (cross), 0.48, 46.8% (radial), 1.65, 73.3% (tangential)	–	[79]
Poplar, PDMS, AgNW ink, Poly (ethylene glycol) diacrylate, 2‐hydroxy‐2‐methyl‐1‐phenyl‐1‐propanone	Delignification, vacuum immersion, ultraviolet irradiation	0.15 × 8 × 8	440	1.01 (0–5 kPa), 0.28 (5–80 kPa), 0.15 (>80 kPa)	46.4	–	5000 (60 kPa)	[89]
Poplar, polydimethlysiloxane	Delignification, freeze drying, vacuum immersion	1 × 10 × 10	1600	0.0584 (tangential) 0.0615(radial) (100–125 kPa), 0.0418 (tangential) 0.0203(radial) (125–520 kPa), 0.027 (tangential) 0.0089(radial) (520–1600 kPa)	2.3	1.3, 4.9% (cross), 0.6, 47.0% (radial), 1.73, 69.4% (tangential)	100 (200 kPa)	[118]

Note: “‐” means not mentioned in the references. Sensitivity is standardized to kPa^−1^, pressure range is standardized to kPa, strength is standardized to MPa for consistency.

### 3.3 Triboelectric Sensor

#### 3.3.1 Principle of Triboelectric Sensor

**Figure 9:**
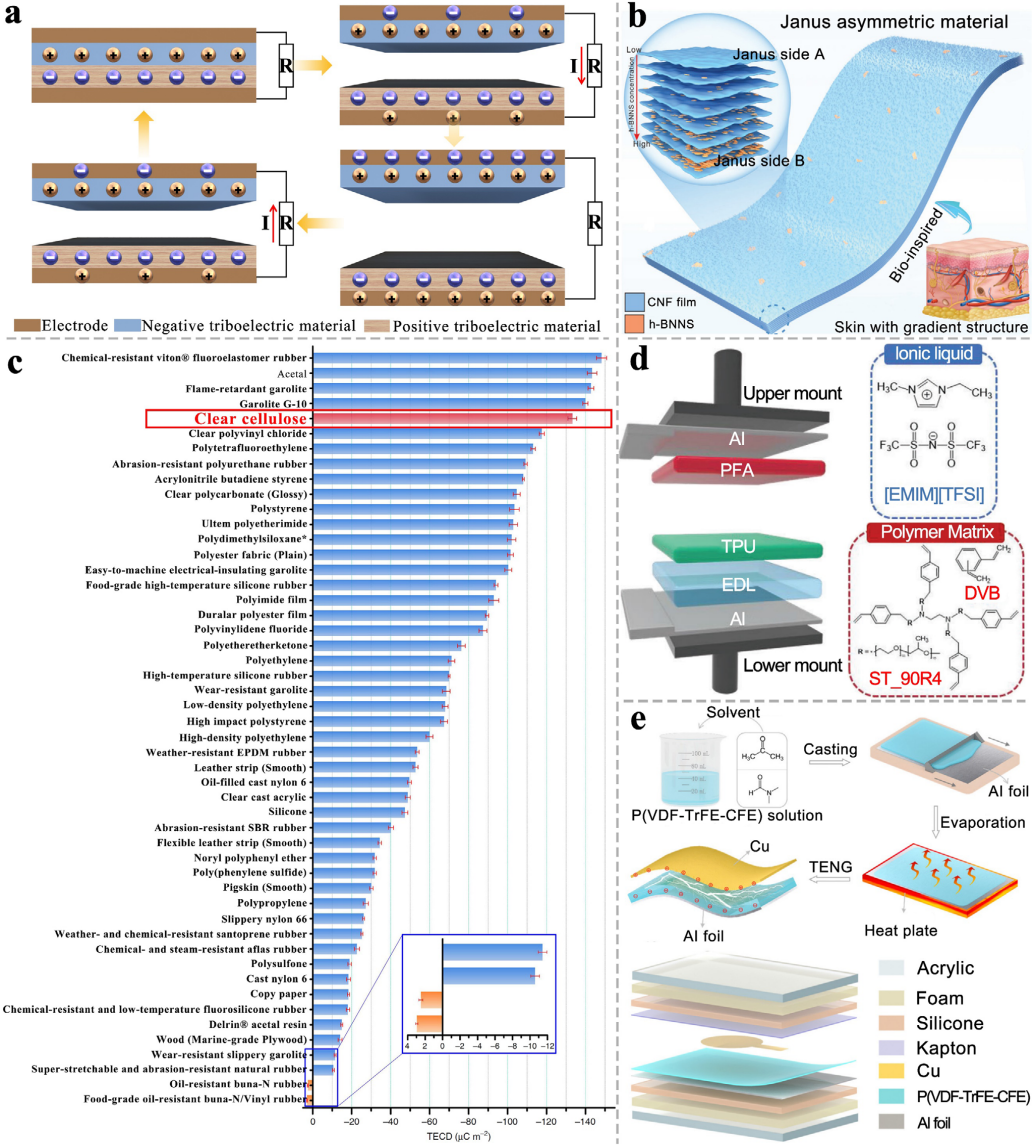
a) Sensing principles of the triboelectric TWPSS. b) A cellulose triboelectric material with an ordered Janus asymmetric design of components and structure. Reproduced with permission.^[^ ^219^ ^]^ Copyright 2025, Wiley‐VCH. c) The quantified triboelectric series. Reproduced with permission.^[^ ^218^ ^]^ Copyright 2019, Springer Nature. d) A TENG that incorporates ion‐containing electrolyte polymer as the intermediate layer. Reproduced with permission.^[^ ^220^ ^]^ Copyright 2024, Wiley‐VCH. e) A TENG with high dielectric permittivity and thin thickness, which can effectively suppress air breakdown. Reproduced with permission.^[^ ^221^ ^]^ Copyright 2024, Royal Society of Chemistry.

When two dissimilar materials come into contact, differences in their electron affinities result in the generation of triboelectric charges at the interface. The material with a greater tendency to lose electrons becomes positively charged, while the one with a higher electron affinity becomes negatively charged. Upon separation, these friction‐induced charges create a potential difference between the top and bottom electrodes, driving electron flow through the external circuit and producing an electric current. When the surfaces recontact, the electrostatically induced charges redistribute through the external load to neutralize the potential difference (**Figure** **9**a). This process stems from the coupled effects of contact electrification and electrostatic induction.^[^ ^209^, ^210^, ^211^ ^]^ The output performance of a triboelectric sensor is governed by various parameters, such as the magnitude of the applied force, contact velocity, interfacial area, and intrinsic material properties. The greater the difference in triboelectric polarity between the two contacting materials, the stronger the output signal.^[^ ^212^ ^]^ Currently, four basic modes of triboelectric nanogenerators (TENG) have been proposed: vertical contact‐separation mode, lateral sliding mode, single‐electrode mode, and independent triboelectric layer mode. Despite structural variations, these modes operate on the same underlying principle.

Cellulose offers significant advantages in triboelectric sensors due to its unique molecular structure. Each repeating unit of cellulose contains three hydroxyl groups; the lone electron pairs of oxygen atoms in these polyhydroxy groups endow cellulose with high electron‐donating ability and a tendency to lose electrons, thereby generating strong triboelectric activity.^[^ ^213^, ^214^ ^]^ The triboelectric contact charge transfer density of cellulose is ≈‐130 µC·m^−2^, and its position in the triboelectric series surpasses that of most common polymers, indicating its potential as a positive‐charge material for triboelectric sensors (Figure 9c).^[^ ^215^, ^216^, ^217^, ^218^ ^]^ In contrast, hemicellulose and lignin occupy intermediate positions in the triboelectric series and exhibit lower charge transfer efficiency. Consequently, to fully harness the triboelectric potential of cellulose in wood‐derived materials, selective removal of hemicellulose and lignin is essential.

Beyond delignification, methods to enhance the output power of triboelectric sensors primarily involve combining wood with materials having stronger electron‐losing ability. Nie et al. introduced an ordered Janus asymmetric design of components and structure, characterized by a transition from low to high dielectric properties from the interior to the surface, accompanied by varying internal charge transfer (Figure 9b).^[^ ^219^ ^]^ This work demonstrates that constructing materials with gradient dielectric properties via different composite methods is a feasible strategy, which can be adopted by TWPSS. In another study, a strategy using ionic electrolyte polymers as the intermediate layer was proposed. By leveraging the high charge capacitance of the ionic electric double layer, this approach effectively mitigates charge decay and maximizes TENG efficiency (Figure 9d).^[^ ^220^ ^]^ Furthermore, designs aimed at suppressing air breakdown in TENG have been reported (Figure 9e).^[^ ^220^ ^]^ Furthermore, designs aimed at suppressing air breakdown in TENG have been proposed (Figure 9e).^[^ ^221^ ^]^ These strategies are crucial for further improving charge density in TENG and merit reference for TWPSS.

#### 3.3.2 Previous Research on Capacitive Sensors

Triboelectric sensors possess several advantages, including a wide variety of material choices,^[^ ^218^, ^222^ ^]^ a simple manufacturing process,^[^ ^223^ ^]^ low cost,^[^ ^224^ ^]^ and spontaneous signal generation.^[^ ^225^ ^]^ Their most prominent advantage lies in their self‐powered capability, which endows them with great potential for applications in ambient energy and bio‐energy harvesting.^[^ ^226^, ^227^, ^228^ ^]^

**Figure 10:**
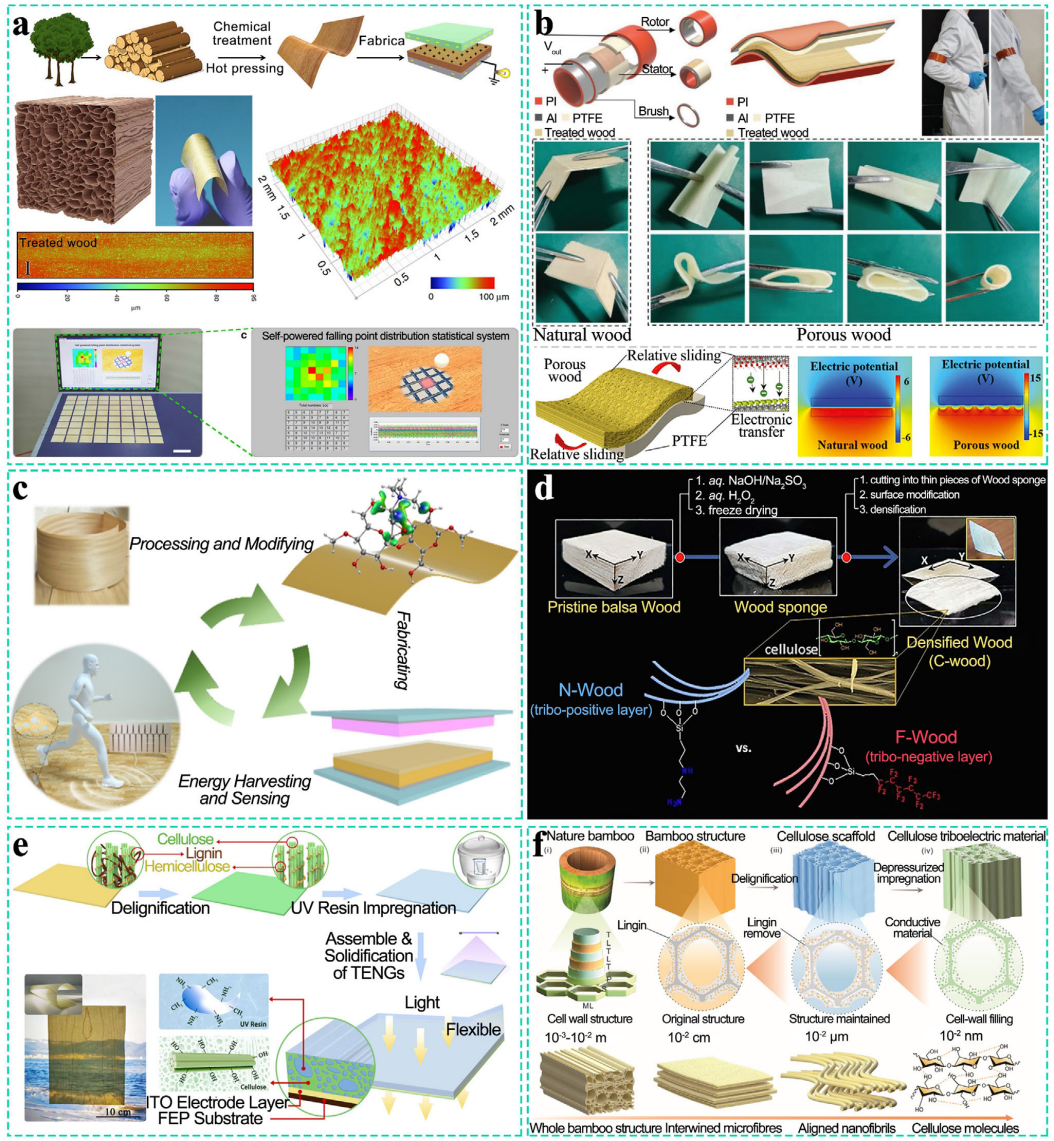
a) A flexible and durable wooden triboelectric sensor obtained through delignification and hot pressing, which is applied in sports training. Reproduced with permission.^[^ ^81^ ^]^ Copyright 2019, Springer Nature. b) A triboelectric sensor featuring a large curvature and ultra‐high stability. Reproduced with permission.^[^ ^117^ ^]^ Copyright 2023, Wiley‐VCH. c) A triboelectric sensor fabricated by cationically modifying the cellulose on the wood surface via immersion in a CHPTAC solution. Reproduced with permission.^[^ ^83^ ^]^ Copyright 2022, American Chemical Society. d) A triboelectric sensor prepared by using wood as both the positive and negative electrodes. Reproduced with permission.^[^ ^230^ ^]^ Copyright 2022, Elsevier. e); Transparent triboelectric TWPSS. Reproduced with permission.^[^ ^93^ ^]^ Copyright 2024, Elsevier. f) The sensor made of bamboo, can maintain high sensing performance after exposure to 200 °C. Reproduced with permission.^[^ ^112^ ^]^ Copyright 2024, American Chemical Society.

Wang's group was the first to propose the preparation of triboelectric materials from natural wood. With the removal of lignin, a large amount of cellulose is exposed, endowing the material with a triboelectric series far superior to that of natural wood and making it suitable as a triboelectric positive material. Subsequent hot‐pressing significantly enhances the tensile strength and wear resistance of this material (**Figure** **10**a).^[^ ^81^ ^]^ This wood‐based triboelectric material, with ultra‐high mechanical strength, has significantly expanded its application scope, enabling its use in scenarios such as flooring. In the single‐electrode mode, a triboelectric sensor was fabricated using this wood as the positive electrode and polytetrafluoroethylene as the negative electrode. The transferred charge density increased by 71% compared to that of natural wood, demonstrating its excellent performance.

Beyond processing techniques, different wood species and microstructures also impact sensor performance. Cao et al. compared the triboelectric properties of four distinct wood species (New Zealand Pine, White Oak, White Beech, and Ash), though their analysis lacked depth.^[^ ^83^ ^]^ Zhang et al. investigated six wood varieties subjected to pretreatment and surface modification, revealing that output performance disparities became more pronounced post‐treatment. They posit that differential adsorption capacities and reactivities toward modifying agents among wood species may accentuate these performance variations following modification. Evidently, discernible differences exist across wood types in both compositional ratios and micromorphological features. Nevertheless, systematic research addressing the influence of wood species on sensor performance remains lacking.^[^ ^229^ ^]^

The above‐mentioned strategies have enhanced the mechanical properties of wood. However, the flexibility of wood sponge has been compromised by the hot‐pressing process. A wood‐based flexible triboelectric sensor with an extremely large curvature was fabricated by omitting the hot‐pressing step, achieving a curvature of 180°. Its compression and bending moduli (362.2 and 220.0 MPa) are far higher than those of natural wood (126 and 96 MPa), which can well meet the requirements for smart wearable devices. Meanwhile, its output power has increased by more than 200% compared to that of natural wood (Figure 10b).^[^ ^117^ ^]^ These works demonstrate the potential of cellulose as a high‐performance positively charged triboelectric material.

To further enhance the electron‐donating capacity of positively charged triboelectric materials, various surface modification strategies—including chemical functionalization and nanostructuring—have been developed. For example, cellulose is cationically modified via an immersion method using a 3‐chloro‐2‐hydroxypropyltrimethylammonium chloride (CHPTAC) solution, where quaternary ammonium groups are introduced into the wood sponge. The triboelectric sensor fabricated from the modified wood sponge exhibits significantly enhanced output performance, nearly six times that of the untreated wood‐based sensor (Figure 10c).^[^ ^83^ ^]^

Meanwhile, negative electrode materials are also being continuously optimized. An all‐wood triboelectric sensor was fabricated using balsa wood as the raw material: the surface of a 2 mm thick wood sponge sheet was modified with trichloro(1H,1H,2H,2H‐perfluorooctyl)silane (AEAP‐Si) and then assembled with unmodified balsa wood, yielding favorable output performance (90.1 V, 114.4 nA·cm^−2^) (Figure 10d).^[^ ^230^ ^]^ Furthermore, a triboelectric sensor utilizing balsa wood as both positive and negative triboelectric materials (with distinct modifications) has been proposed: balsa wood subjected to “ammonia modification (N‐(2‐aminoethyl)‐3‐aminopropyltrimethoxysilane)” and “fluorine modification (trichloro(1H,1H,2H,2H‐perfluorooctyl)silane)” serves as the positive and negative electrode materials, respectively. This sensor exhibits excellent mechanical properties, flexibility, and electrical performance. These studies provide insights into the preparation of all‐wood triboelectric materials.^[^ ^230^ ^]^

The above studies still predominantly focus on cellulose, modifying it to achieve superior triboelectric performance. In contrast, other research endeavors employ wood as a framework, integrating high‐triboelectric‐potential materials (e.g., PTFE, PVDF) onto its surface or into its interior. For example, wood modified by in situ growth of zeolitic imidazolate framework‐8 (ZIF‐8) becomes more triboelectrically positive. Meanwhile, the increased surface roughness of ZIF‐8‐modified wood enhances the sensor's sensitivity. Similarly, wood coated with PDMS becomes more triboelectrically negative. A triboelectric nanogenerator fabricated using these two modified materials can generate an open‐circuit voltage and short‐circuit current that are 80 times higher than those of natural wood under a 50 N force.^[^ ^231^ ^]^ Expanding on wood‐derived materials, Huang et al. attempted to use a wood‐derived carbon electrode to fabricate a single‐electrode triboelectric sensor.^[^ ^113^ ^]^

Building upon the basic sensing function, triboelectric TWPSS integrated with additional characteristics have also been proposed. Wu introduced a method entailing delignification and impregnation with ultraviolet curable resin. This approach enabled the attainment of an optical transmittance as high as 88.8% in wood. Moreover, when this treated wood was utilized as the raw material to fabricate the triboelectric TWPSS, its electrical output performance was enhanced by 6.5‐fold compared to that of natural wood (Figure 10e).^[^ ^93^ ^]^ Another type of transparent wood combined with epoxy resin (containing amino and methyl groups) has achieved a 530% improvement in output performance (≈127 V).^[^ ^232^ ^]^ The transparent wood prepared by Chu et al. not only exhibits high output performance but is also endowed with antibacterial properties, expanding its applicability in scenarios requiring hygiene control (e.g., medical environments).^[^ ^94^ ^]^

As wood is among the most prevalent materials for interior decoration, triboelectric sensors that integrate the natural patterns of wood and possess sensing capabilities exhibit promising application prospects in indoor settings. Simultaneously, fire prevention in indoor spaces represents an indispensable and crucial aspect that cannot be overlooked. The fire resistance of wood‐based triboelectric materials can be significantly enhanced through the in situ growth of flame retardants and the formation of a dense char layer. This leads to a reduction of 95.4% in the heat release rate (HRR) and 94.2% in the total heat release rate (THR). Such improvements are essential for indoor fire safety, particularly for the large‐scale application of triboelectric TWPSS in public spaces.^[^ ^233^ ^]^

Similarly, bamboo—another lignocellulosic material—has been utilized for performance optimization. In one study, continuous conductive pathways were formed by loading carbon nanotubes (CNTs) into the pores of delignified bamboo. The sensor based on this triboelectric material exhibits excellent sensitivity of 33.61 kPa^−1^ within the range of 0–2.25 kPa. It maintains high sensing performance even after exposure to 200 °C and can provide accurate feedback on the human body's motion state (Figure 10f).^[^ ^112^ ^]^

However, the output performance of triboelectric sensors is constrained by the electron‐donating capability of cellulose. Even after modifications such as cationic functionalization, there remains an upper limit to the output voltage/current. Furthermore, the surface roughness of wood is difficult to control with high precision, leading to insufficient signal consistency among batches during mass production. Although hot‐pressing treatment can enhance wear resistance, repeated friction still tends to damage the surface structure, reduce charge transfer efficiency, and compromise long‐term performance. The raw materials, manufacturing processes, and properties of triboelectric TWPSS are summarized in **Table** **6**.

**Table 6:** Raw materials, fabrication processes, and performance of triboelectric TWPSS.

Positive electrode	Preparation method	Negative electrode	Mode	Contact area [mm]	Frequency [Hz]	Output voltage, current [V, µA]	Transferred charge density [µC·m^−2^)	Power density [mW·m^−2^]	Lord [MΩ]	Stability [cycle tests]	Response time, Recovery time [ms, ms]	Ref.
Balsa wood	delignification, hot pressing	PTFE	Single electrode	30 × 30	1	81, 1.8 (13.3 kPa stress, 20 N)	36	57	40	20 000	<25	[81]
New Zealand pine	delignification, freeze drying	PTFE	Single electrode	80 × 80	2	220 ± 20, 5.8 ± 0.5	–	158.2	50	500	–	[229]
Balsa wood, PFOT‐Si	delignification, freeze drying, immersion	Balsa wood, AEAP‐Si	Vertical contact separation	20 × 20	3	90.1, 0.458 (8.2 N)	–	–	4.7	5000	–	[230]
Norway spruce, balsa wood, European yew, ZIF‐8	in situ growth ZIF‐8	Norway spruce, balsa wood, European yew, PDMS	Vertical contact separation	35 × 20	–	24.3,0.32 (50 N)	12	10.4	80	1500	–	[231]
Balsa wood	delignification, hot pressing	PTFE	Vertical contact separation	20 × 20	1	38, 0.37 (2.5 kPa)	–	–	–	100 000	<50	[82]
Balsa wood, CHPTAC	delignification, immersion, pressing	PTFE	Single electrode	20 × 20	5	335, 9.74 (2.5 kPa)	71.45	3.8	200	20 000	–	[83]
Balsa wood	delignification	PTFE	Vertical contact separation	35 × 35	4	45, 1.5 (2.5 kPa)	0.1	–	≈5	–	30, 210	[117]
Balsa wood, 3‐Aminopropyltriethoxysilane	delignification, vacuum immersion	Balsa wood, PFDTMS	Vertical contact‐separation	40 × 50	0.5, 1.0, 1.5	31.27, 0.34 (5.4 MPa)	–	–	–	20 000	–	[234]
Eucalyptus wood	delignification, freeze drying	FEP	Vertical contact separation	12 × 30	5‐	208, 60	‐	4.86	5.7	10 000	–	[113]
Maple	delignification, vacuum immersion, ultraviolet light	FEP	Vertical contact separation	50 × 50	1	212, ‐	–	43.7	90	10 000	–	[93]
Poplar, methacrylic anhydride, 4‐Dimethylaminopyridine	delignification, immersion	PU foam	Single electrode	35 × 35	3	29.3, 1.04	52.4	5.88	5	10 000	46, 67	[94]
Maple, Epoxy resin	delignification, vacuum immersion	FEP	Vertical contact separation	40 × 40	1	127, 0.5 (10 N)	–	–	–	10 000	–	[232]
Pine wood, H_9_N_2_O_4_P, P_2_O_5_, C_3_H_6_N_6_, CNTs	delignification, freeze drying, growth preparation, drying, vacuum immersion, hot pressing	FEP	Vertical contact‐separation	10 × 10	–	90, ‐ (250 °C)	–	–	9	15 000	72, 63	[233]
Moso bamboo/CNTs	delignification, freeze drying, high pressure immersion, freeze drying	FEP	Vertical contact‐separation	40 × 10	–	43, ‐	–	–	–	10 000	36, 45	[112]

Note: “‐” means not mentioned in the references. Output voltage is standardized to V, output current is standardized to µA for consistency.

### 3.4 Piezoelectric Sensor

#### 3.4.1 Principle of Piezoelectric Sensor

**Figure 11:**
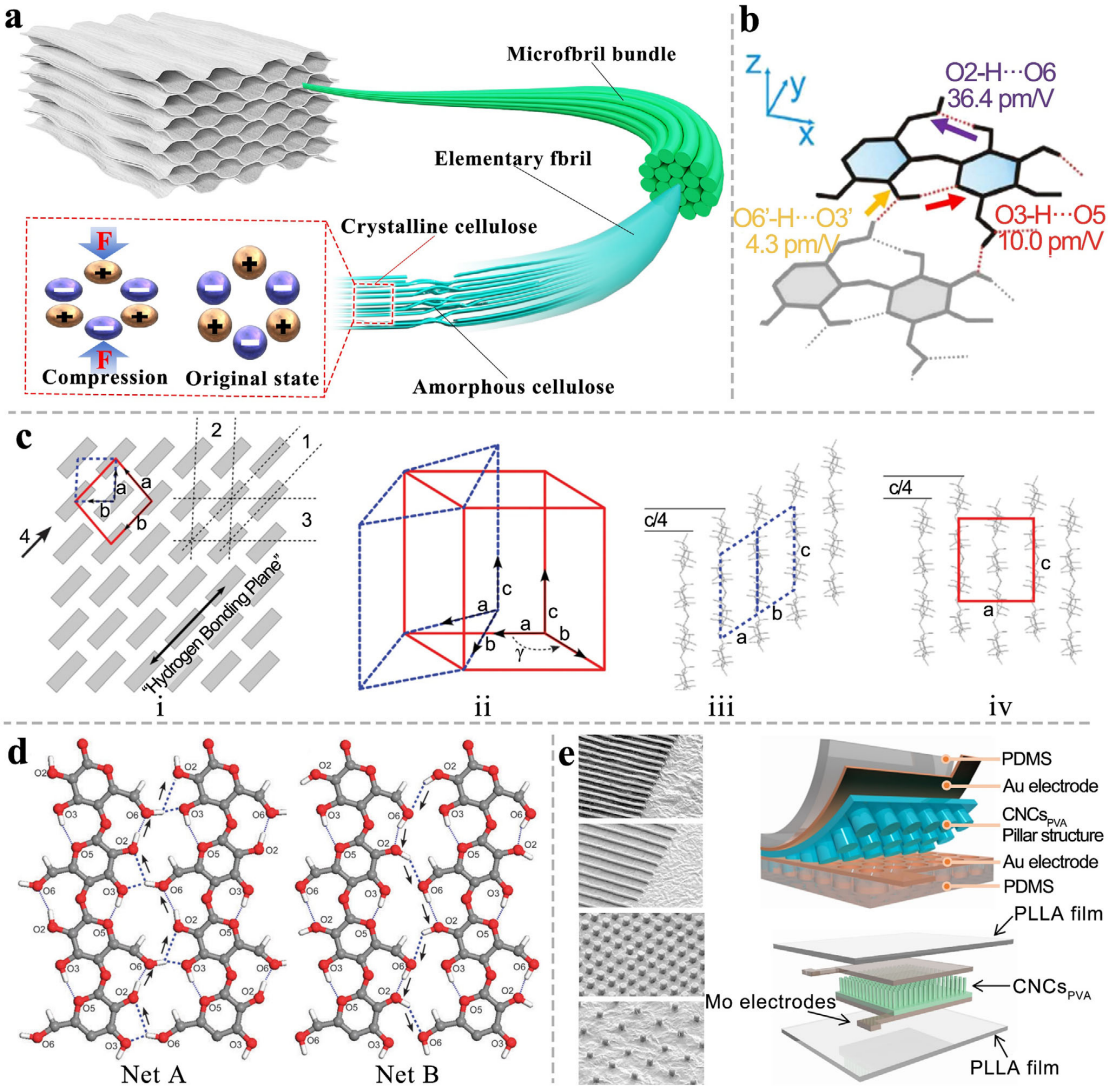
a) Sensing principles of the piezoelectric TWPSS. b) Schematic of the unit cells for cellulose *I*_α_ (triclinic, dashed line) and *I*_β_ (monoclinic, solid line). Reproduced with permission.^[^ ^241^ ^]^ Copyright 2023, American Chemical Society. c) Schematic diagram of cellulose crystal structure: projection along the cellulose chain with the asymmetrical triclinic Iα and monoclinic Iβ structure. Reproduced with permission.^[^ ^240^ ^]^ Copyright 2011, Royal Society of Chemistry. d) Schematics of two networks A, B within the hydrogen‐bonded plane, (110)_t_ and (200)_m_. Reproduced with permission.^[^ ^242^ ^]^ Copyright 2008, American Chemical Society. e) A cellulose nanocrystals piezoelectric sensor assembled in multilayer piezoelectric materials through submicrometer patterning. Reproduced with permission.^[^ ^243^ ^]^ Copyright 2025, Springer Nature.

Piezoelectric sensors primarily consist of piezoelectric active materials capable of converting mechanical stimuli into electrical energy. Upon application of external stress, deformation of directionally aligned non‐centrosymmetric crystal structures induces spatial separation of positive and negative charges. This results in the accumulation of charges on opposing electrodes, enabling the generation and transmission of electrical signals.^[^ ^235^, ^236^ ^]^ Over 30% of known materials worldwide exhibit the piezoelectric effect. Wood, as a widely available natural resource, is among the most abundant piezoelectric materials. Its piezoelectricity arises from permanent dipole moments generated by the intrinsic asymmetry of its crystalline structure and the highly ordered hydrogen‐bond network in cellulose (**Figure** **11**a).^[^ ^237^, ^238^, ^239^ ^]^

Cellulose I crystals exist in two polymorphs depending on the extraction source: the triclinic structure (Iα) and the monoclinic structure (Iβ), neither of which possesses a center of symmetry (Figure 11c).^[^ ^240^ ^]^ Within each unit, three types of hydrogen‐bond interactions occur between O2‐H and O6, O3‐H and O5, and O3‐H and O6, respectively. These hydrogen bonds are all strong dipoles, with piezoelectric responses calculated via density functional theory (DFT) of 36.4, 10, and 4.3 pm·V^−1^, respectively (Figure 11b).^[^ ^241^ ^]^ Additionally, cellulose molecular chains are connected via inter‐chain hydrogen bonds within the (110) and (200) planes, forming two coexisting hydrogen‐bond networks (Figure 11d).^[^ ^242^ ^]^ This enables polar hydrogen bonds in non‐centrosymmetric order to exhibit a net dipole moment and thus piezoelectric activity.^[^ ^240^ ^]^

However, the overall piezoelectric response is significantly reduced due to the compensation effect of adjacent hydrogen bonds in the crystal and the mechanical contraction of covalent bonds. For instance, the longitudinal piezoelectric coefficient d33 of cellulose paper is only 0.4 pC·N^−1^.

The intrinsically weak piezoelectric properties of natural cellulose pose challenges for the development of high‐performance wood‐based piezoelectric sensors. **Table** **7** summarizes the piezoelectric coefficients—including the transverse piezoelectric constant (d31) and longitudinal piezoelectric constant (d33)—of high‐performance piezoelectric materials, wood, and wood‐based composites, highlighting this performance gap. Nevertheless, the hierarchical architecture and unique structural features of wood offer advantages for enhancing piezoelectric activity.

**Table 7:** Piezoelectric coefficients of high‐performance piezoelectric materials, wood, and wood‐based composite materials.

Piezoelectric materials	Structure	Piezoelectric coefficient		Refs.
		d31 [pC/N]	d33 [pC/N]	
BaTiO3	Bulk	–	75−190	[244]
BaTiO3	Thin film	−34.5	85.6	[245]
BaTiO3	Ceramic	−79	191	[246]
ZnO	Nanorods	–	11.8	[247]
ZnO	Bulk	5	12.4	[248]
PZT	Nanofibers	–	500‐600	[249]
PZT	Nanowires	–	152	[250]
Japanese cypress	Slice	−0.00145	–	[251]
Long leaf pine	Slice	0.00203	–	[251]
Carpinus tschonoskii	Slice	0.002	–	[251]
Acer mono	Slice	0.00118	–	[251]
Wood impregnated with Rochelle salt	Slice	–	11	[252]
Wood microfibers embedded in PDMS	Slice	–	11 ± 2.3	[253]
Wood microfibers hybrid (50 wt.%) with BaTiO3	Slice	–	5	[253]
Cellulose paper	Film	–	0.4 ± 0.08	[244]
CNF	Film	–	5.7 ± 1.2	[237]

Note: “‐” means not mentioned in the references.

A large body of research has employed bottom‐up approaches, extracting cellulose from natural wood to fabricate piezoelectric cellulose papers and related functional materials (Figure 11e).^[^ ^216^, ^243^ ^]^ In contrast, top‐down fabrication of wood‐derived piezoelectric materials is less common but leverages the special dynamic responses of naturally locked cellulose crystals within wood. In the macroscopic wood matrix, cellulose crystals are more easily displaced under small loads, significantly improving the piezoelectric generation efficiency.

#### 3.4.2 Previous Research on Piezoelectric Sensors

Owing to the intrinsic piezoelectric properties of cellulose crystals, charges are generated when the wood sponge is subjected to compression, and these charges can be collected on its surface. The higher compressibility of the wood sponge induces greater displacement of crystalline cellulose, thereby enhancing electrical output performance. Based on this principle, a wood sponge with piezoelectric effects can be prepared by subjecting balsa wood to a simple delignification treatment (using a mixture of hydrogen peroxide and acetic acid) followed by freeze‐drying. Under a constant stress of 13.3 kPa, the piezoelectric output of this wood sponge is significantly improved compared to that of the original wood, with an increase of more than 85‐fold.^[^ ^76^ ^]^

**Figure 12:**
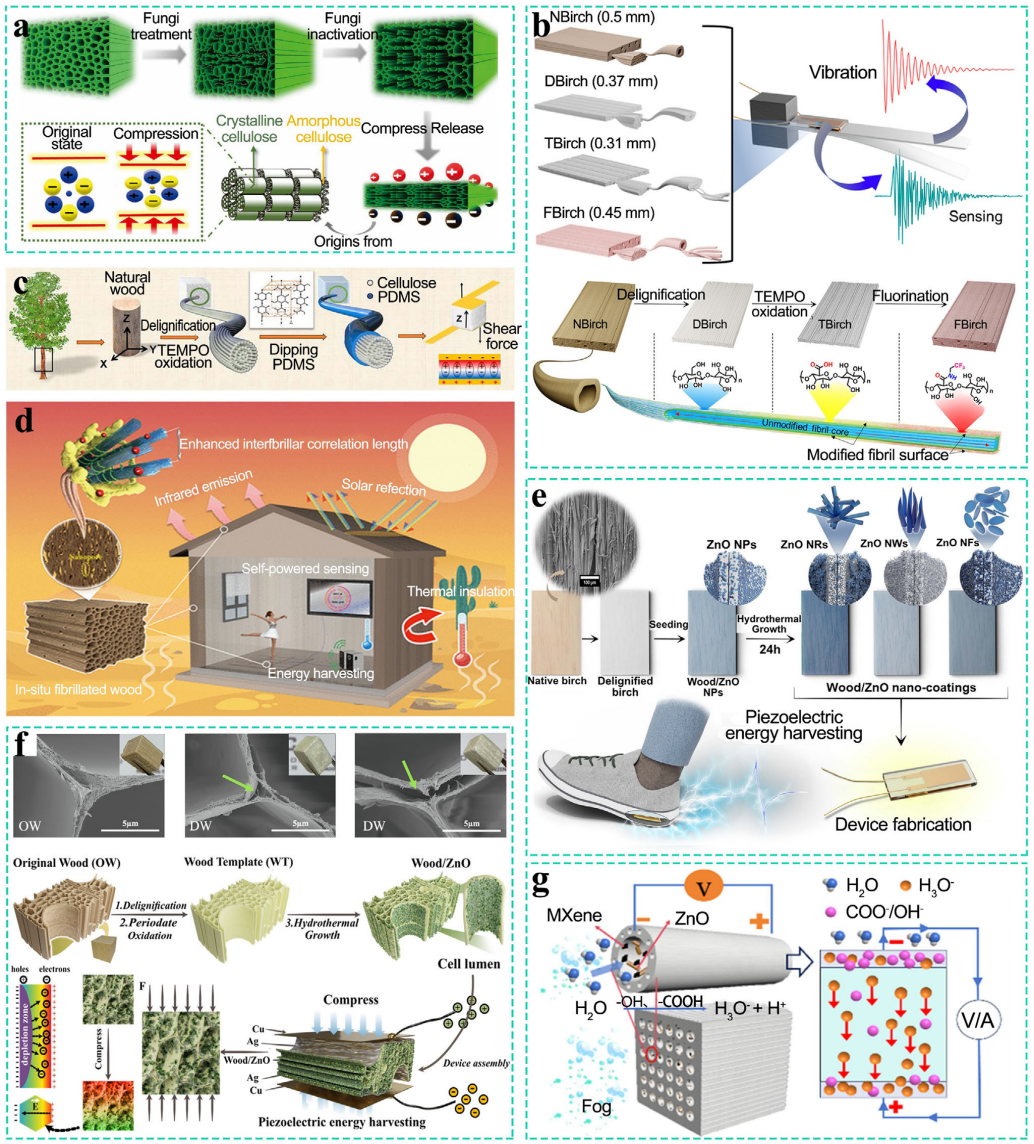
a) Fungal decay pretreatment (wood sponge). Reproduced with permission.^[^ ^69^ ^]^ Copyright 2021, American Association for the Advancement of Science. b) Sequential delignification, oxidation, and model fluorination (preserves native crystalline piezoelectric cellulose). Reproduced with permission.^[^ ^90^ ^]^ Copyright 2022, American Chemical Society. c) TEMPO/NaBr/NaClO oxidation combined with pressure impregnation. Reproduced with permission.^[^ ^107^ ^]^ Copyright 2023, American Chemical Society. d) Deep eutectic solvent treatment (TWPSS) Reproduced with permission.^[^ ^254^ ^]^ Copyright 2024, American Chemical Society. e) Oxidation and model fluorination (wood sponge). Reproduced with permission.^[^ ^91^ ^]^ Copyright 2022, Elsevier. f) Hydrothermal growth of ZnO surface coating (high piezoelectric output). Reproduced with permission.^[^ ^92^ ^]^ Copyright 2023, Elsevier. g) Incorporation for multifunctional (piezoelectric/moist‐electric) composites. Reproduced with permission.^[^ ^255^ ^]^ Copyright 2024, Elsevier.

To achieve a more environmentally friendly preparation process, the previous chemical delignification scheme was replaced with an eco‐friendly and sustainable fungal decay pretreatment. For example, a decayed wood cube (15 × 15 × 13.2 mm) with 45% mass loss during decay exhibits an output voltage more than 55‐fold higher than that of the original wood. However, the fungal decay pretreatment is time‐consuming (requiring 10 weeks) and thus challenging for scalable, efficient production (**Figure** **12**a).^[^ ^69^ ^]^ Additionally, this method risks reducing the mechanical properties and durability of the wood.

Another strategy for preparing wood‐based piezoelectric materials involves baking wood under a nitrogen atmosphere. This process selectively removes most hemicellulose and part of the lignin while retaining crystalline cellulose fibers, resulting in a safe wood‐based piezoelectric material (free of chemical residues). While preserving the stability of the wood's micro‐nano structure, this strategy significantly enhances its shape recovery ability. This enables cellulose microfibers to displace more easily under slight loads, thereby strengthening the piezoelectric effect. After this treatment, the output voltage and current of the wood exceed 70‐fold those of natural wood.^[^ ^115^ ^]^

However, relying solely on the piezoelectric effect of cellulose, its performance still lags significantly behind that of traditional high‐performance piezoelectric materials (e.g., ZnO). Modification of wood is a common strategy to enhance its piezoelectric performance. After birch veneers undergo delignification, 2,2,6,6‐tetramethylpiperidine‐1‐oxyl (TEMPO) oxidation, and trifluoroethylamine fluorination, their piezoelectric performance is enhanced at the molecular and nanoscale. This improvement is associated with the increased spacing of nanofibers induced by delignification and the enhanced local deformation of individual cellulose fibrils (Figure 12b).^[^ ^90^ ^]^ To further optimize performance, a composite strategy combining TEMPO/NaBr/NaClO oxidation with impregnation has been developed. Specifically, the wood sponge is subjected to controlled compression during impregnation with PDMS, which synergistically improves its mechanical properties and electromechanical conversion efficiency.

After the wood sponge is oxidized, the primary alcohol hydroxyl groups on the fibers are selectively oxidized to carboxyl groups, thereby increasing the electrostatic repulsion between the fibers. The oxidant acts on the amorphous regions and parts of the crystalline regions, which increases the crystallinity of cellulose and thus enhances piezoelectric performance. In addition, PDMS possesses a permanent electric dipole moment and can form hydrogen bonds with cellulose, which significantly increases the density of electric dipoles, endowing the wood sponge with more prominent piezoelectric performance (Figure 12c).^[^ ^107^ ^]^

Jiang et al. proposed a new strategy that disrupts the hydrogen bond network in the cell wall while carboxylating the wood components, without significantly altering the natural hierarchical structure of the wood. The treated wood exhibits ultra‐high compressive strength and cyclic stability, which enhances the displacement of cellulose crystals under mechanical stress. Consequently, the generated piezoelectric output is 400 times higher than that of natural wood (Figure 12d).^[^ ^254^ ^]^

The aforementioned studies primarily focus on utilizing the intrinsic properties of cellulose in wood or modifying cellulose fibers to enhance wood's piezoelectric performance. Another strategy to improve the piezoelectric performance of wood‐based materials involves using wood as a template and filling its surface or interior with materials exhibiting excellent piezoelectric properties. ZnO is frequently chosen as a research target due to its high piezoelectric performance and the ease of controlling its morphology during synthesis. However, ZnO suffers from poor compressibility and tends to agglomerate during preparation, which limits its direct application as a piezoelectric material. In contrast, the porous structure of wood provides an ideal template to address these issues.

In specific preparation methods, the hydrothermal growth method can produce nanoscale ZnO with various morphological structures on the wood surface by precisely controlling the growth time. Experimental results indicate that the output voltage of TWPSS fabricated via this strategy is highly sensitive to the morphology of its nanostructures. Specifically, nanowires and nanorods with a high aspect ratio can generate high output voltages even at low contents (Figure 12e).^[^ ^91^ ^]^ Beyond surface growth, attempts have also been made to hydrothermally grow ZnO inside wood. Specifically, oxidizing wood with sodium periodate increases the number of charges on the fiber surface, promoting the rapid diffusion of Zn^2^⁺ into the negatively charged wood sponge and its binding to carboxyl or aldehyde groups within the sponge. This process enables the uniform distribution of ZnO inside the wood (including in cell lumens and cell walls). Compared to surface‐only modification, this strategy significantly enhances the piezoelectric performance of wood‐based piezoelectric materials (Figure 12f).^[^ ^92^ ^]^

Yuan et al. developed a wood‐based material integrating both piezoelectric and hygroelectric properties. ZnO was loaded onto the wood sponge via hydrothermal growth, yielding high ion diffusion efficiency. Additionally, when metal oxides such as ZnO come into contact with water molecules in humid air, a streaming current is generated (Figure 12g).^[^ ^255^ ^]^ Notably, ZnO is not the only filler material explored. Rochelle salt, a food additive, has also been investigated. It offers environmental advantages, and its crystal growth inside wood occurs spontaneously.^[^ ^252^ ^]^

For piezoelectric wood‐derived pressure/strain sensors prepared via the top‐down approach, the primary task is to expose cellulose with high piezoelectric activity. This operation not only improves the compressibility of wood but also provides sufficient reaction sites and high reactivity for subsequent modification treatments. However, compared with traditional piezoelectric ceramics and other materials, the piezoelectric performance of natural cellulose remains relatively low. To address this, a series of improvement methods have been proposed, including optimizing the preparation process, implementing chemical modification, and incorporating nanoscale fillers, with the aim of enhancing output performance—a core theme in this research field.

Certain piezoelectric materials (e.g., ZnO) exhibit excellent linear deformation characteristics. Researchers have attempted to utilize such materials to improve the performance of wood‐derived piezoelectric sensors while enhancing the linearity of their output signals. Theoretically, the uniaxial alignment of cellulose crystals and an increase in the crystallinity index can enhance piezoelectric performance. However, the top‐down preparation method inherently limits the feasibility of achieving uniaxial alignment of cellulose crystals. Furthermore, the current understanding of cellulose crystallinity and surface properties remains insufficient, which impairs the reproducibility of fabricated piezoelectric devices and hinders their subsequent application and scaling‐up. This has become a key factor restricting the development of this field. The raw materials, manufacturing processes, and properties of piezoelectric TWPSS are summarized in **Table** **8**.

**Table 8:** Raw materials, processes, and performance of piezoelectric TWPSS.

Main material	Preparation method	Size: thickness× length× width [mm]	Output voltage, current [V, µA]	Stability [cycle tests]	Refs.
Fir wood, Rochelle salt	immersion, dry	2.3 ‐ 3.3 (thickness), diameter 22	–	–	[252]
Balsa wood	delignification, freeze drying, oven drying	15 × 15 × 14	0.69, 0.0071 (13.3 kPa stress)	≥ 600	[76]
Balsa wood	oven drying, fungal treatment, oven drying	15 × 15 × 13.2	0.87, 0.0133 (45 kPa stress); 1.32, 0.019 (100 kPa stress)	500	[69]
Birch wood, TEMPO, NaBr	delignification, immersion, immersion	0.5 × 50 × 20	0.06, ‐	5000	[90]
Birch wood, ZnO_2_	delignification, seeding, and hydro thermal growth	0.5 × 50 × 20	1.3–1.4, ‐ (3.25 cm^2^ 2.46 kPa stress)	≈ 1000	[91]
Balsa wood, sodium periodate, ZnO_2_	delignification, hydro thermal growth	10 × 10 × 15	1.5, 0.00291 (8–10N stress)	300	[92]
Balsa wood, TEMPO, NaBr, Silver epoxy adhesive	delignification, vacuum immersion	1 × 10 × 10	2.88, 0.21009	–	[107]
Balsa wood, Imidazole, Triethylmethylammonium Chloride	immersion, freeze drying	10 × 11 × 12	2, 0.00576 (221 kPa, 1.7 Hz)	2000	[254]
Balsa wood	delignification, roasted	15 × 15 × 15	1.4, 0.0145	1000	[115]
Balsa wood, polyvinylpyrrolidone, Na_2_TeO_3_	delignification, carbonization, immersion	15 × 15 × 15	–	500	[114]
Balsa wood, Zn(NO_3_)_2_⋅6 H_2_O, TEMPO, PDMS, Ti_3_AlC_2_	delignification, oxidation, impregnation	10 × 10 (6 × 6)	4.21, 0.020285	10 000	[255]

Note: “‐” means not mentioned in the references. Output voltage is standardized to V, output current is standardized to µA for consistency.

## 4 Applications

Wood‐derived sensors have demonstrated remarkable potential across diverse application domains owing to their exceptional properties, including biodegradability, renewable sourcing, and tunable mechanical/electrical sensitivity. The practical implementation of these advanced sensing materials spans various fields such as wearable electronics, environmental monitoring, and smart agriculture.^[^ ^256^ ^]^

### 4.1 Healthcare and Human Motion Detection

**Figure 13:**
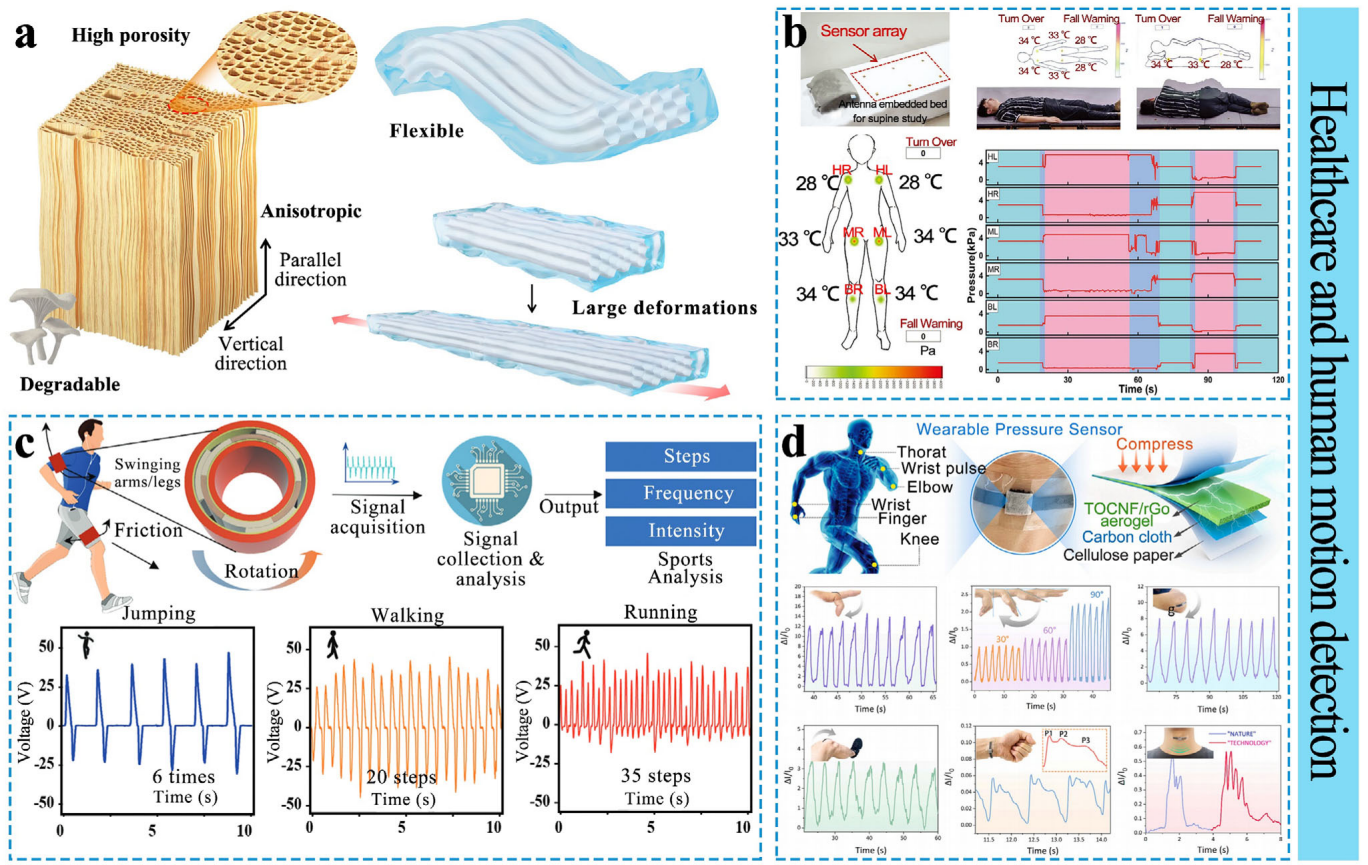
a) Wood exhibits inherent characteristics including anisotropy, high porosity, and biodegradability. Wood‐derived hydrogel materials demonstrate properties such as bendability and large deformation capability. b) Temperature and pressure sensors for monitoring bedsores. Reproduced with permission.^[^ ^121^ ^]^ Copyright 2023, Wiley‐VCH. c) A sensor with a large curvature for detecting the human body's motion state. Reproduced with permission.^[^ ^117^ ^]^ Copyright 2023, Wiley‐VCH. d) A highly sensitive sensor that can be used to detect throat vibrations and pulse. Reproduced with permission.^[^ ^258^ ^]^ Copyright 2023, Elsevier.

Wood‐derived sensors, particularly hydrogels incorporating the delignified wood sponge framework, exhibit strong suitability for healthcare and human motion detection due to their inherent biocompatibility, biodegradability, and self‐adhesion.^[^ ^257^ ^]^ The low modulus and high compressibility of the wood sponge enable these sensors to conform comfortably to the skin, allowing for accurate detection of subtle physiological signals such as pulse waves and throat vibrations with high sensitivity. This deformability, inherited from wood's hierarchical porous structure, further enhances their adaptability to complex human movements. For applications requiring monitoring of specific directional strains (e.g., finger bending, elbow flexion), the inherent anisotropy of the wood sponge structure can be strategically oriented to optimize signal response along the desired axis. **Figure** **13**a summarizes the characteristics of wood and hydrogel, including anisotropy, biodegradability, and flexibility. Figure 13a presents the characteristics of wood and wood‐derived hydrogel, including anisotropy, biodegradability, and flexibility, etc.

In conclusion, wood‐derived sensors can detect strains generated by joint bending (e.g., fingers, elbows, and knees) to assess human physical states (Figure 13c,d).^[^ ^88^, ^97^, ^99^, ^117^, ^258^ ^]^ They also exhibit capability in speech recognition: the sensor can detect and distinguish subtle differences in laryngeal vibrations induced by different words (Figure 13d).^[^ ^101^, ^165^ ^]^ For bedridden patients, a flexible, multi‐parameter, passive, wireless, and battery‐free sensor fabricated from fir wood is utilized to reduce the risk of pressure ulcers in hospitalized patients. It enables temperature monitoring with a resolution of 0.1 °C, while the sensor array can track patient posture, providing a novel approach for caring for long‐term bedridden patients (Figure 13b).^[^ ^121^ ^]^ Currently, these applications remain relatively simplistic, focusing primarily on basic flexion‐extension movements and vibrations. The subsequent data analysis and feedback have not yet fully met practical needs, necessitating further research.

### 4.2 Smart Home

**Figure 14:**
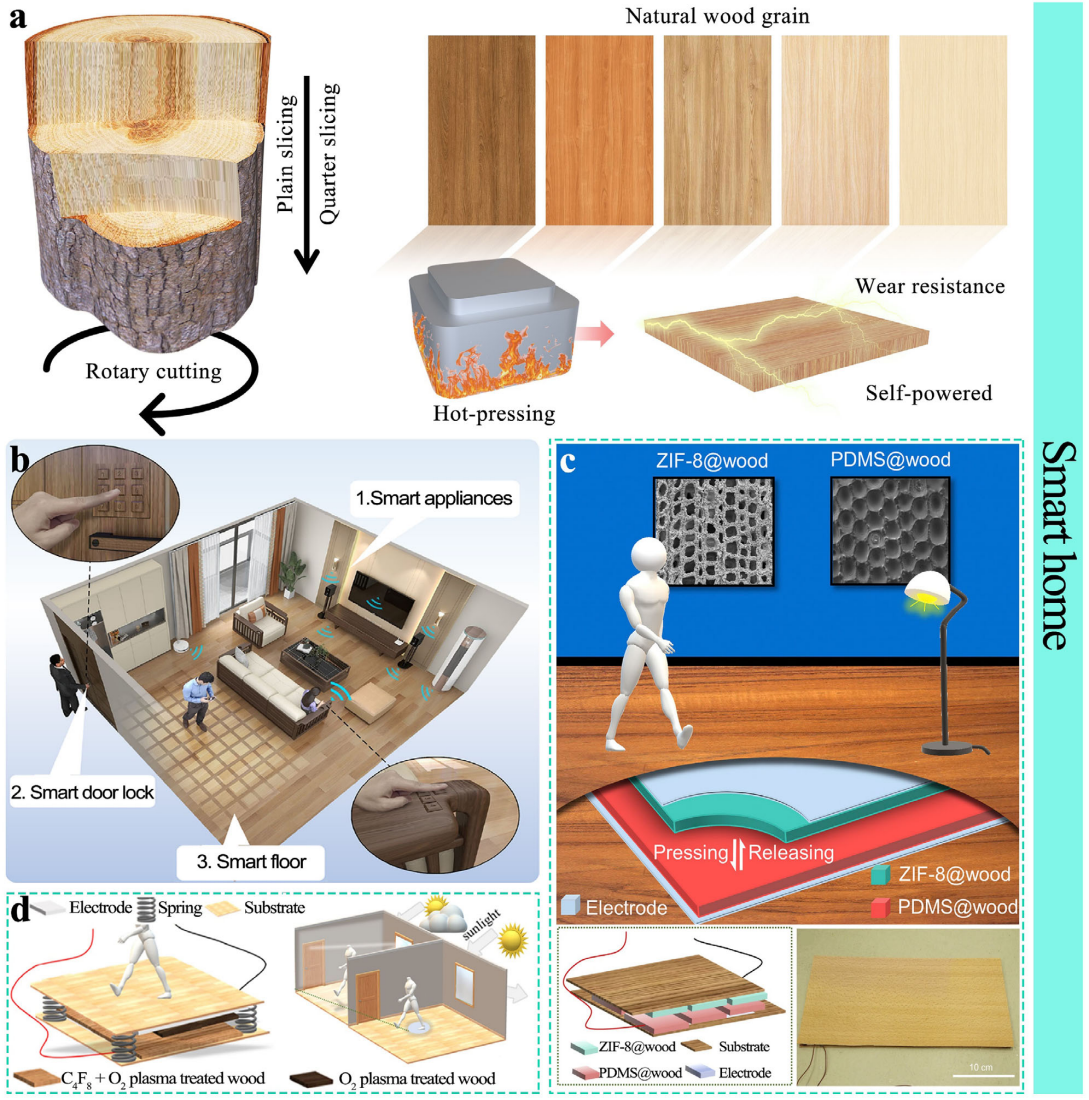
a) Wood veneers exhibiting natural grain patterns are obtained through rotary cutting, plain slicing, and quarter slicing. Hot‐pressing densified wood demonstrates enhanced wear resistance and serving as functional components in triboelectric self‐powered sensors. b) Triboelectric sensors are used for the control of electrical appliances in smart homes, smart door locks, and the monitoring of human motion states. Reproduced with permission.^[^ ^82^ ^]^ Copyright 2022, American Chemical Society. c,d) Triboelectric sensor generators supply power to electrical appliances. Reproduced with permission.^[^ ^231^, ^259^ ^]^ Copyright 2021; Copyright 2021, Elsevier.

The natural aesthetics and texture of wood are largely preserved in sensors fabricated via top‐down processing (through controlled delignification) (**Figure** **14**a). This characteristic enables seamless integration into furniture and architectural elements such as floors or tabletops without compromising interior design. Such aesthetic compatibility, combined with wood's inherent structural integrity, constitutes a unique advantage for unobtrusive smart home integration.

Triboelectric sensors fabricated from densified delignified wood (e.g., through hot‐pressing) retain or even exceed the mechanical strength of natural wood. Consequently, these sensors exhibit sufficient durability for high‐traffic applications like smart flooring. Moreover, wood‐based triboelectric sensors possess self‐powering capabilities, thereby eliminating external battery requirements and simplifying deployment within smart home networks.

Based on these properties, triboelectric self‐powered sensors can be integrated with furniture to control electrical appliances and lighting fixtures. They can also enable smart door locks with a dual‐security mechanism combining personal characteristics and passwords. Smart floors incorporating such sensors can detect movement trajectories and monitor fall safety. These sensors feature low cost, convenient operation, environmental friendliness, and energy efficiency (Figure 14b–d)^[^ ^82^, ^83^, ^231^, ^259^, ^260^ ^]^ However, current research remains limited to matrix arrangements and combinations of single‐type sensing devices (e.g., flooring), with each device offering only a single function. Thus, a significant gap remains from the realization of a truly intelligent home.

### 4.3 Judgment and Training in Sports

The abundant void structure and high strength of wood endow it with exceptional specific strength (Figure 14a). Simultaneously, the use of low‐density materials such as balsa wood ensures that integrated sensors add minimal weight to sports equipment, thus preserving its performance. Furthermore, under cyclic loading, densified delignified wood (processed via hot‐pressing) exhibits superior elastic recovery and fatigue resistance (Figure 14a), making it particularly suitable for sensors in sports equipment subjected to repetitive impacts.

**Figure 15:**
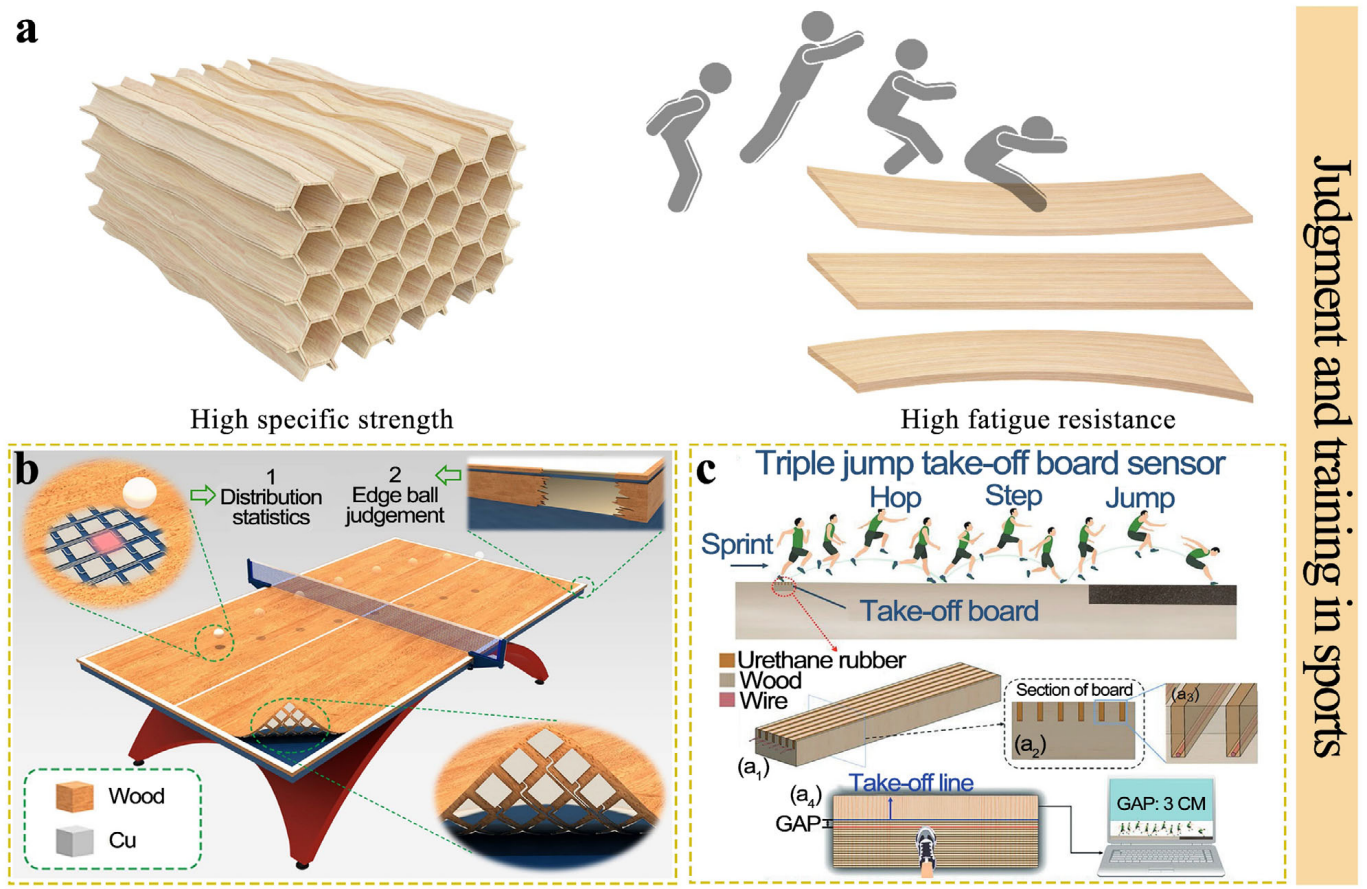
a) The void structure of wood endows it with inherent attributes of low density and high specific strength. Densified delignified wood exhibits superior elastic recovery and fatigue resistance. b) The sensor is used for a table tennis. Reproduced with permission.^[^ ^81^ ^]^ Copyright 2019, Springer Nature. c) The sensor is used for determining the results of the triple jump. Reproduced with permission.^[^ ^19^ ^]^ Copyright 2022, Elsevier.

Triboelectric sensors, particularly those operating in single‐electrode mode, offer advantages of structural simplicity and self‐powering capability. This eliminates the need for external power sources, rendering them highly beneficial for sports‐related applications. Luo et al. fabricated a wood‐based triboelectric nanogenerator (TENG) for smart table tennis tables. It is completely self‐powered by friction, capable of converting table tennis impact energy into electrical signals while enabling speed sensing and movement path tracking (**Figure** **15**b).^[^ ^81^ ^]^ Xu et al. proposed a smart self‐powered takeoff board based on triboelectric sensors, which enables precise detection of triple jump athletes’ takeoff states with an accuracy of up to 1 mm (Figure 15c).^[^ ^261^ ^]^ Based on these advantages, wood‐based materials hold potential for applications in table tops or floors of gymnastics, basketball, and volleyball courts, facilitating athlete training and competition monitoring.

## 5 Conclusion and Outlook

This review systematically summarizes recent progress in top‐down fabricated wood‐derived pressure/strain sensors (TWPSS), with a focus on key aspects including material selection, structural design, sensing mechanisms, and applications. While leveraging wood's natural hierarchical structure and sustainability confers unique advantages, critical challenges persist, alongside promising research directions to address these limitations.

### 5.1 Current Challenges

The performance of TWPSS requires careful balancing. For instance, achieving high sensitivity—typically dependent on low modulus and large deformation—inherently compromises the working range and linearity. Beyond this fundamental trade‐off, enhancing the output power of piezoelectric TWPSS remains a critical hurdle. Their performance still lags significantly behind that of conventional piezoelectric materials (e.g., ZnO, PVDF), limiting their viability for self‐powered applications.

Beyond these electrical performance limitations, wood's inherent structural heterogeneity poses a severe challenge to batch‐to‐batch consistency and reproducibility, hindering standardization and mass production. Additionally, a comprehensive life cycle assessment is essential to evaluate environmental sustainability. Although wood is a renewable resource, the current chemical delignification process involves reagent consumption, energy input, and wastewater generation—factors that may undermine the environmental advantages of biomaterials.

Addressing these environmental concerns alone is insufficient for real‐world deployment, as current lab‐scale processes (e.g., vacuum impregnation, freeze‐drying, manual assembly) are inherently incompatible with mass production. Finally, reliable packaging and seamless integration of TWPSS with essential components (power sources, signal conditioning circuits, and wireless communication modules) present significant challenges, particularly regarding interface compatibility and long‐term reliability.

### 5.2 Future Directions

Future research should actively design and fabricate biomimetic micro/nano architectures within wood. Techniques such as ice‐templating with controlled gradients could create structures (e.g., gradient porosity) to decouple the sensitivity‐range‐linearity trade‐off.

Exploring the sensing mechanisms of TWPSS, particularly hybrid mechanisms, could unlock new performance boundaries. For example, combining piezoresistive and piezoelectric effects in a single device enables decoupled detection of static and dynamic stimuli.^[^ ^110^ ^]^ Additionally, leveraging wood's intrinsic properties (e.g., thermal insulation from porous structures) could lead to multifunctional sensors (e.g., pressure‐temperature dual‐parameter detection).^[^ ^97^, ^98^, ^99^, ^106^ ^]^

Deep Eutectic Solvents (DES) hold immense promise as greener alternatives for delignification, but challenges such as slow diffusion kinetics and inefficient solvent recovery remain to be addressed. Developing alternatives to freeze‐drying (e.g., supercritical CO_2_ drying[264,265]) is critical for reducing energy consumption while preserving the porosity of wood sponges.

Establishing a universal benchmark for pressure/strain sensors fabricated from biomaterials is crucial for fair comparison with other sustainable sensors, such as cellulose‐based bottom‐up devices or chitosan‐based sensors.

### 5.3 Broader Inspiration

The material strategies and processing insights related to TWPSS can accelerate the development of fully biodegradable circuits, displays, and energy storage devices, providing a practical solution to the global electronic waste crisis.

Low cost and biodegradability make TWPSS ideal for ubiquitous environmental monitoring networks. Smart wood‐derived sensors, capable of analyzing indicators such as soil moisture in forests or farmland, harmlessly degrade after use, aligning with the goals of sustainable agriculture and ecosystem protection.

Cellulose's biocompatibility and biodegradability position TWPSS as distinctive candidates for next‐generation biomedical devices. They show significant promise for long‐term implantable sensors, smart wound dressings, and other clinical applications.

Given that lignin can be selectively and quantitatively removed, TWPSS is capable of preserving the natural wood texture. Consequently, it can be seamlessly and aesthetically integrated into smart homes (e.g., sensor floors, responsive furniture) and wearables (e.g., fabric‐integrated sensors), advancing smart living environments while maintaining natural aesthetics.

In conclusion, TWPSS represents a compelling intersection of sustainability and functionality, leveraging wood's natural advantages to address the environmental challenges of conventional electronics. Overcoming current bottlenecks—from performance trade‐offs to scalable fabrication—will require interdisciplinary collaboration across materials science, environmental engineering, and electronics. By providing a blueprint for green sensing technologies, this review aims to inspire innovations not only in TWPSS but also in broader fields striving for a more sustainable and interconnected world.

## Conflict of Interest

The authors declare no conflict of interest.
